# Integration of whole-exome sequencing and structural neuroimaging analysis in major depressive disorder: a joint study

**DOI:** 10.1038/s41398-024-02849-4

**Published:** 2024-03-09

**Authors:** Eun-Young Oh, Kyu-Man Han, Aram Kim, Youbin Kang, Woo-Suk Tae, Mi-Ryung Han, Byung-Joo Ham

**Affiliations:** 1 Division of Life Sciences, College of Life Sciences and Bioengineering, Incheon National University, Incheon, Republic of Korea; 2 Department of Psychiatry, Korea University Anam Hospital, Korea University College of Medicine, Seoul, Republic of Korea; 3 Brain Convergence Research Center, Korea University College of Medicine, Seoul, Republic of Korea; 4 Department of Biomedical Sciences, Korea University College of Medicine, Seoul, Republic of Korea

**Keywords:** Diagnostic markers, Depression

## Abstract

Major depressive disorder (MDD) is a common mental illness worldwide and is triggered by an intricate interplay between environmental and genetic factors. Although there are several studies on common variants in MDD, studies on rare variants are relatively limited. In addition, few studies have examined the genetic contributions to neurostructural alterations in MDD using whole-exome sequencing (WES). We performed WES in 367 patients with MDD and 161 healthy controls (HCs) to detect germline and copy number variations in the Korean population. Gene-based rare variants were analyzed to investigate the association between the genes and individuals, followed by neuroimaging-genetic analysis to explore the neural mechanisms underlying the genetic impact in 234 patients with MDD and 135 HCs using diffusion tensor imaging data. We identified 40 MDD-related genes and observed 95 recurrent regions of copy number variations. We also discovered a novel gene, *FRMPD3*, carrying rare variants that influence MDD. In addition, the single nucleotide polymorphism rs771995197 in the *MUC6* gene was significantly associated with the integrity of widespread white matter tracts. Moreover, we identified 918 rare exonic missense variants in genes associated with MDD susceptibility. We postulate that rare variants of *FRMPD3* may contribute significantly to MDD, with a mild penetration effect.

## Introduction

Major depressive disorder (MDD) is a common psychiatric disorder and has a significant impact on an individual’s quality of life, including both social and economic aspects [1]. MDD is characterized by the occurrence of a distinct depressive episode lasting for a minimum of 2 weeks, accompanied by significant changes in mood, interest, and enjoyment [2]. Both genetic and environmental factors play key roles in the etiology of MDD, with estimated genetic inheritance rates up to 25% for a severe recurrent MDD [3]. Common variants account for ~21% of the genetic effects of MDD [4], and rare variants with a moderate penetration effect may contribute to the underlying genetic causes of MDD through additional inheritance mechanisms [5, 6].

Genome-wide association studies (GWASs) have played a crucial role in unraveling the genetic basis of complex diseases and quantitative traits [7]. These studies systematically assess common variants, usually with a minor allele frequency (MAF) > 5% [8]. Despite these findings, much of the genetic contribution to complex traits remains undisclosed, even in diseases where large GWAS meta-analyses have been undertaken [9, 10]. Consequently, many studies have been proposed to elucidate the genetic causes of complex diseases from the perspective of “missing heritability” [11–13]. Recent studies using next-generation sequencing (NGS) suggest that rare variants (MAF < 1%) are associated with complex diseases [14–16]. Thus, rare variants analyses would be promising to clarify additional disease risks or trait variability.

Although studies have indicated a shared genetic basis between common variants associated with lifetime MDD and depressive symptoms in the general population, it remains unclear whether this association applies to rare variants [17]. Advancement in NGS technology can help identify rare variants [18]. Previous studies have conducted gene-based analyses of rare damaging variants to identify genes related to MDD using the UK Biobank exome dataset [19, 20]. Zhou et al. revealed that genes based on rare variants, including *FOXO1*, *MAPK10*, *DLGAP3*, *ARID5B*, *ASXL2*, and *MED13*, were significantly associated with MDD [19]. Cheng et al. performed a gene-based burden test to identify *OR8B4*, *TRAPPC11*, *SBK3*, and *TNRC6B* between patients with MDD with high polygenic risk scores (PRS) and those with low PRS [20].

The pathophysiology of MDD can be characterized by the dysfunction of brain networks involved in emotional regulation, reward processing, and cognitive controls [21, 22]. White matter tract-based structural connectivity provides a physical and structural basis for these psychopathology-related brain networks [23]. A growing body of evidence has shown microstructural abnormalities in white matter tracts measured by diffusion tensor imaging (DTI) in patients with MDD [24, 25]. For example, a recent study using DTI data from 1305 patients with MDD and 1602 healthy controls from 20 samples worldwide reported MDD-related lower fractional anisotropy (FA) and higher radial diffusivity (RD) in the widespread white matter tracts [26]. White matter microstructures, as measured by DTI, exhibit generally high genetic heritability [27–29]. Moreover, several studies have suggested that genetic contributions to MDD may be mediated by genetically heritable variations in white matter microstructures [30, 31]. A recent study using data from the UK Biobank of 6,401 individuals reported that the PRS for MDD obtained from a previous genome-wide association study (GWAS) was correlated with lower FA and higher MD in several white matter tracts [30].

An approach combining WES and neuroimaging phenotypes in MDD could provide deeper insights into the heritable neural architecture related to MDD and related genetic variants [32]. However, only a very small number of studies using both DTI data and a hypothesis-free genetic approach (i.e., PRS or WES) have been conducted in patients with MDD [33]. Thus, in the present study, we aimed to combine WES and DTI data in patients with MDD to investigate the correlation between genetic variants and abnormalities in white matter structural connectivity using a relatively large sample compared to previous WES studies on MDD [32]. Among the widely studied neuroimaging phenotypes, we opted for tractography-based parameters of white matter structural connectivity. This choice is grounded in the observation that white matter microstructures, as measured by DTI, generally exhibit high genetic heritability, and few studies have investigated genetic correlations using a hypothesis-free approach in MDD [33].

In this study, we hypothesized that the complex genetic inheritance patterns of MDD could be attributed to rare variants with moderate effects. Moreover, we expected that single nucleotide polymorphisms (SNPs)—particularly those associated with neurobiological pathways involved in neural plasticity and brain development – from the case-control association analysis would be associated with lower FA and AD (axial diffusivity) and higher RD and MD (mean diffusivity) in the white matter tracts, including the superior longitudinal fasciculus (SLF), inferior longitudinal fasciculus (ILF), forceps major (FMajor), forceps minor (FMinor), and uncinate fasciculus (UNC). This hypothesis was based on results from previous studies utilizing hypothesis-free genetic approaches on DTI parameters in MDD [30, 31]. To investigate these hypotheses, we performed WES to analyze the gene-based rare variants and examined the potential correlations between SNPs and the DTI parameters reflecting white matter structural connectivity in patients with MDD.

## Materials and methods

### Sample participants

A total of 367 patients with MDD were recruited from outpatient psychiatric clinics at Korea University Anam Hospital, Seoul, Republic of Korea, from March 2010 to February 2021. Patients with MDD were aged 19 years or older, and their diagnoses were confirmed by board-certified psychiatrists (B.-J.H. and K.-M.H.) using the Structured Clinical Interview from the Diagnostic and Statistical Manual of Mental Disorders, Fourth Edition, for Axis I disorders through a full psychiatric assessment. The exclusion criteria for the MDD group were as follows: (i) the presence of any other significant psychiatric disorder, (ii) MDD with psychotic features, (iii) acute suicidal tendencies requiring immediate inpatient care, (iv) a history of a severe medical illness, (v) primary neurological disorders (e.g., Parkinson’s disease, cerebrovascular disease, or epilepsy), and (vi) contraindications for magnetic resonance imaging (MRI). Board-certified psychiatrists used the life-chart methodology to assess the total illness duration. For the healthy control (HC) group, a total of 161 participants who were 19 years or older were recruited from the community with advertisements. Full psychiatric assessments were conducted by the board-certified psychiatrists for the HCs, and none of them had any current or past psychiatric disorders. The exclusion criteria mentioned above were also applied to the HCs. Within the entire sample, 234 patients and 135 HCs underwent MRI and were included in the analysis of neuroimaging parameters. The detailed demographic and clinical characteristics for the neuroimaging analyses are presented in Table 1. After the MRI scan, we used the 17 item Hamilton Depression Rating Scale developed by Hamilton in 1960 to evaluate the severity of depressive symptoms in all participants [34]. All participants were confirmed to have Korean ancestry within the past three generations via self-reports. The study protocol was approved by the Institutional Review Board of the Korea University Anam Hospital (2009AN0105, 2015AN0009, 2016AN0213, 2017AN0185, and 2019AN0174). Prior to inclusion in this study, all participants provided written informed consent according to the principles outlined in the Declaration of Helsinki. Notably, statistical methods were not employed to predetermine the sample size. Additionally, randomization was not performed in the experiments, and the investigators were not blinded to assignment during both the experiments and outcome assessments.

**Table 1:** Demographic and clinical characteristics of patients with major depressive disorder and healthy controls.

Characteristics	MDD	HC	*P-*value (*t*, *χ*^*2*^)
**Total sample**			
*N*	367	161	NA
Age	40.79 ± 14.17	39.62 ± 14.30	0.068 (*t* = 1.8273)
Sex (Female/Male)	243 (66.2%) / 124 (33.8%)	103 (64.0%) / 58 (36.0%)	0.619 (*χ*^*2*^ = 0.248)
Education years	13.11 ± 3.19	15.04 ± 2.26	<0.001 (*t* = −7.374)
HDRS-17 score	16.39 ± 6.65	0.93 ± 1.69	<0.001 (*t* = 41.070)
Remission state / depressive state	40 (10.9%) / 327 (89.1%)	NA	NA
Illness duration (months)	29.02 ± 31.22	NA	NA
**Neuroimaging sample**	**MDD**	**HC**	***P*-value (*t***, ***χ***^***2***^**)**
*N*	234	135	NA
Age	40.53 ± 14.25	37.57 ± 14.24	0.055 (*t* = 1.925)
Sex (Female/Male)	158 (67.5%) / 76 (32.5%)	83 (61.5%) / 52 (38.5%)	0.240 (*χ*^*2*^ = 1.378)
Education years	13.05 ± 3.24	15.07 ± 2.26	<0.001 (*t* = −7.061)
HDRS-17 score	14.95 ± 6.90	1.00 ± 1.79	<0.001 (*t* = 29.266)
Remission state / depressive state	36 (15.4%) / 198 (84.6%)	NA	NA
Illness duration (months)	30.26 ± 32.59	NA	NA
TICV (cm^3^)	1436.99 ± 150.87	1465.01 ± 154.01	0.089 (*t* = −1.706)
Drug-naive / Drug-treated patients (n)	73 (31.2%) / 161 (68.8%)	NA	NA
Medication, n			
**Antidepressants**			
SSRI	64 (39.7%)	NA	NA
SNRI	39 (24.2%)		
NDRI	4 (2.5%)		
NaSSA	9 (5.6%)		
Others	8 (5.0%)		
Combination of AD	37 (23.0%)		
**Antipsychotics**			
None	109 (67.7%)		
AP	40 (54.8%)		
Combination of AP	12 (7.5%)		

Data are mean ± standard deviation for age, education years, HDRS-17 scores, illness duration, and TICV.

*P*-values for sex distribution were obtained using the chi-squared test.

*P*-values for comparisons of age, years of education, HDRS-17 scores, and TICV were obtained using an independent *t*-test.

*HC* healthy controls, *MDD* patients with major depressive disorder, *HDRS-17* 17-item Hamilton Depression Rating Scale, *TICV* total intracranial cavity volume, *SSRI* selective serotonin reuptake inhibitor, *SNRI* serotonin and norepinephrine reuptake inhibitor, *NDRI* norepinephrine-dopamine reuptake inhibitor, *NaSSA* noradrenergic and specific serotonergic antidepressant, *combination of AD* a combination of two or more types of antidepressants, *APs* antipsychotics, *combination of AP* a combination of two or more types of antipsychotics.

### WES and processing

Genomic DNA was obtained from the peripheral blood of patients with MDD (*n* = 367) and HCs (*n* = 161) (Table 1); the Agilent SureSelect Human All Exome V5 kit (Agilent Technologies, Santa Clara, CA, USA) was used according to the manufacturer’s instructions. WES with 101 bp paired-end reads was performed on a HiSeq2000, HiSeq2500, or HiSeq4000 (Illumina, San Diego, CA, USA).

After sequencing, the quality of the raw data was assessed using FastQC (https://www.bioinformatics.babraham.ac.uk/projects/fastqc/; v0.11.9). Trimmomatic (v0.36) [35] was used to remove low-quality fragments and adapter sequences. Burrows-Wheeler Aligner-Maximal Exact Match (BWA-MEM, v0.7.17-r1188) [36] was used to align reads on the GRCh38 reference genome. The Genome Analysis Toolkit (GATK, v4.2.0.0) [37] was used for marking duplicates, local realignments, and recalibration scoring based on GATK Best Practices. For downstream analysis, aligned reads with low mapping quality (MAPQ < 20) were removed using SAMtools (v1.10) [38].

### Germline variants calling and filtering in MDD-related genes

Germline short-variant discovery was performed following GATK Best Practices (v4.2.0.0) [37]. Briefly, insertions and deletions (INDELs) and SNPs were detected using *HaplotypeCaller* in the GVCF mode. Joint genotyping was performed using *GenomicsDBImport* and *GenotypeGVCFs* to identify potential variants at the individual level. *SelectVariants* and *VariantFilteration* were run to select and filter the SNPs and INDELs based on the following criteria: QD < 2.0 | | FS > 200.0 || ReadPosRankSum < −20.0. Germline variants within the coding sequences were selected and their functional consequences, including silent or non-silent variants, were predicted using (annotate variation; ANNOVAR) [39]. To extract MDD-related genes, a systematic literature review was performed using the following terms: (“major depressive disorder” OR “major depression” OR “depression” OR “depressive”) AND (“WES” OR “WGS”) in English-language peer-reviewed journals published up to October 2022 using the PubMed database. In total, 21 articles were selected and after conducting manual screening of the abstracts and titles, only 5 out of 21 articles were used to identify novel MDD-related genes for further analyses.

### Germline copy number alterations (CNAs)

To detect germline CNAs, CNVkit (v0.9.9) [40] was used for 367 patients with MDD based on a hidden Markov model approach. Germline CNAs were filtered using the default parameters to decrease the number of false-positive segments. Subsequently, significant amplifications and deletions of the chromosomal arms and focal regions across patients with MDD were identified using Genomic Identification of Significant Targets in Cancer 2.0 (GISTIC2.0) [41] on the segmentation data produced by the CNVkit.

### Case-control association analysis of common SNPs

Germline variants were filtered by the following criteria using PLINK (v1.07) [42]: excluding SNPs with the Hardy-Weinberg equilibrium exact test (*P* < 0.001), eliminating samples with call rates below 95%, and excluding SNPs with call rates below 95%. A dominant logistic regression model was used to identify the common SNPs associated with MDD.

### Gene-based rare variant association analysis

We performed four gene-based rare variant analyses between patients with MDD and HCs, including combined multivariate and collapsing (CMC) [43], variable threshold model by permutation (VT) [44], sequencing kernel association test (SKAT) [45], and optimal sequencing kernel association test (SKATO) [46] using Rvtests (v20190205) [47]. We applied principal component analysis (PCA) to condense the genetic burden information associated with the genomic regions using the FastPCA algorithm [48]. Rvtests (v20190205) [47] were run with 1,000,000 permutations and covariates including age, sex, and two principal components. Rare coding variants with a minor allele count ≥ 3 were tested for associations under two MAF thresholds (MAF ≤ 1% and MAF ≤ 0.1%) and two functional categories (non-synonymous and damaging). Variants were annotated using ANNOVAR [39]. Additionally, we used the aggregated Cauchy association test (ACAT) to boost the statistical power of our analysis by combining all four test results, as described by Liu et al. [49]:

$${P}_{ACAT}=\frac{1}{4}\mathop{\sum }\limits_{i=1}^{4}tan\{(0.5-{p}_{i})\pi \},$$

where *p*_*i*_ is the *p*-value of the test (CMC, VT, SKAT, or SKATO); the four tests are regarded equally in the combination.

### MRI data acquisition and imaging processing

234 patients with MDD and 135 HCs from the total sample underwent DTI using a 3.0 Tesla Trio™ whole-body MR scanner (Siemens Healthcare GmbH, Erlangen, Germany) at the Korea University MRI Center. The detailed DTI parameters are described in the Supplementary Material. For white matter tract analysis, four DTI parameters, FA, MD, RD, and AD, from the 18 white matter tracts were automatically calculated using the Tracts Constrained by Underlying Anatomy (TRACULA) developed by Yendiki et al. [50] implemented in the FreeSurfer 7.2 version (Laboratory for Computational Neuroimaging, Athinoula A. Martinos Center for Biomedical Imaging, Charlestown, MA, USA; http://surfer.nmr.mgh.harvard.edu). TRACULA reconstructs 18 major white matter pathways in the bilateral hemispheres using automated global probabilistic tractography processes and DTI data [51–53]. In the present study, all four complementary parameters were used in the analysis.

The 18 major white matter tracts are as follows [50]: FMajor and FMinor of the corpus callosum, anterior thalamic radiation (ATR), cingulum-angular bundle (CAB), cingulum-cingulate gyrus bundle (CCG), corticospinal tract (CST), ILF, superior longitudinal fasciculus-parietal bundle (SLFp), superior longitudinal fasciculus-temporal bundle (SLFt), and UNC.

### Statistical methods for neuroimaging-genetic association analysis

For the neuroimaging-genetic association analysis, we investigated the association between significant SNPs and structural connectivity of white matter tracts and applied three-step analyses as follows. First, the SNPs were selected from two categories: (i) results of case-control association analysis (*P* < 0.001) and (ii) variants with high mutation frequency (≥20%) in samples from MDD-related genes. Second, to compare the neuroimaging markers between the two groups, a one-way analysis of covariance (ANCOVA) was performed, including the diagnostic group (i.e., MDD versus (vs.) HC) as an independent variable; the extracted values of neuroimaging markers (i.e., four DTI parameters on 18 white matter tracts) as dependent variables; and age, sex, and years of education as covariates. Third, the association between the SNPs and neuroimaging markers was investigated in the MDD and HC groups. Two-way ANCOVA was used to examine the effects of genotype (i.e., a dominant model that compares non-risk allele homozygotes to risk allele carriers) or genotype-by-diagnosis interactions on neuroimaging markers with the following variables: (i) four DTI parameters of 18 major white matter pathways as dependent variables; (ii) genotypes and diagnosis (i.e., MDD vs. HC) as independent variables; and (iii) age, sex, and years of education as covariates. To prevent type I errors, the Bonferroni method was used for multiple comparisons in the neuroimaging-genetic association analysis.

## Results

### Profiles of germline variants and MDD-related genes

In this study, we analyzed 367 MDD and 161 HC genomes to detect germline variants. The mean sequencing depth was 117.7X (47.4–148.8X) for all samples. Using WES, we obtained an average of 44,633 variants (42,730–52,439 variants per sample; median, 43,968). On average, 9686 non-silent (9426–11,897 per sample; median, 9671) and 10,141 silent (9890–12,278 per sample; median, 10,132) variants were identified in patients with MDD. The most common point mutations were T > G (21%) and T > C (20.7%).

**Fig. 1:**
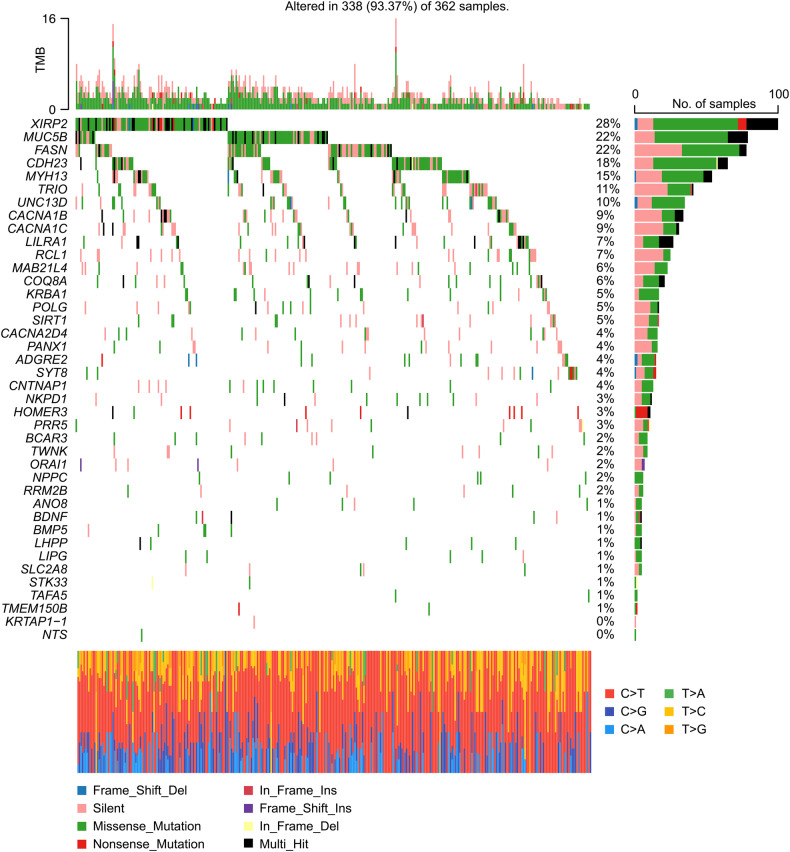
Mutational landscape of the major depressive disorder (MDD) group. The left-side plot displays mutated MDD-related genes (rows) across patients with MDD (columns), featuring the 40 genes from the 44 MDD-related genes based on mutation frequency across patients. Four out of the 44 MDD-related genes were not identified in our MDD patients. On the right side, a bar plot shows the number of mutated patients in the MDD group. Percentages indicate the proportion of patients with an identified mutation in each gene. The stacked bar plot on the bottom shows the distribution of the single nucleotide variants classified into six transition and transversion events for each sample. “Multi-Hit” denotes that more than one mutation was detected in a gene within one patient.

After the systematic literature review, a total of 44 MDD-related genes were identified, and 40 of the 44 genes were found in the current study in 367 patients with MDD (Supplementary Table S1) [4, 5, 32, 54–62]. Rare exonic missense variants (with MAF <1%) of these genes were selected using the 1000 Genomes Project data, the Korean Variant Archive [63], and the Genome Aggregation Database. Of the 367 patients with MDD, 338 had 918 rare exonic missense variants in 40 MDD-related genes. The top five most frequently mutated genes were *XIRP2* (28%), *MUC5B* (22%), *FASN* (22%), *CDH23* (18%), and *MYH13* (15%), as shown in Fig. 1.

### Recurrent CNAs

**Fig. 2:**
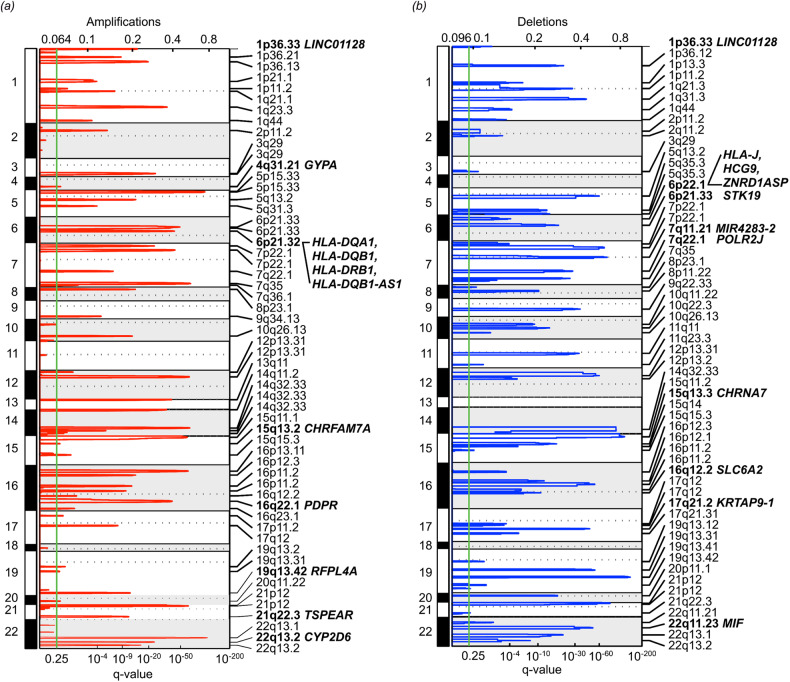
Recurrent copy number alterations (CNAs). **a** Recurrent focal amplified regions (red line) and **b** deleted regions (blue line) detected by GISTIC 2.0 analysis in 367 patients with major depressive disorder (MDD) and 161 healthy controls. The horizontal axis represents the *q-*value and the vertical axis represents the chromosome number. MDD-related genes are highlighted in bold-face. The green lines represent the threshold for significance (*q*-value < 0.25).

We detected CNAs in patients with MDD and in HCs to identify recurrent CNA regions. Forty-five recurrent focally amplified CNA regions and 50 recurrent focally deleted regions were identified in MDD genomes (Fig. 2). Copy number gains at 21p12 (40%) and losses at 21p12 and 15q11.2 (43% observed in both cases) were the most recurrent CNA regions.

Recurrent focal amplification regions with MDD-related genes were detected at 1p36.33 (*LINC01128*), 4q31.21 (*GYPA*), 6p21.32 (*HLA-DQA1, HLA-DQB1, HLA-DRB1*, and *HLA-DQB1-AS1*), 15q13.2 (*CHRFAM7A*), 16q22.1 (*PDPR*), 19q13.42 (*RFPL4A*), 21q22.3 (*TSPEAR*), and 22q13.2 (*CYP2D6*) (Fig. 2a). Recurrent focal deletion events with MDD-related genes were found at 1p36.33 (*LINC01128*), 6p22.1 (*HLA-J, HCG9*, and *ZNRD1ASP*), 7q11.21 (*MIR4283-2*), 7q22.1 (*POLR2J*), 15q13.3 (*CHRNA7*), 16q12.2 (*SLC6A2*), 17q21.2 (*KRTAP9-1*), and 22q11.23 (*MIF*) (Fig. 2b). In addition, the sizes of the CNAs annotated as MDD-related genes are provided in Supplementary Table S2.

### Gene-based rare variants analysis

We performed CMC, VT, SKAT, and SKATO tests to identify genes associated with MDD using two MAF thresholds (“MAF ≤ 1%” or “MAF ≤ 0.1%”) and functional categories of “damaging” or “nonsynonymous.” The CMC method collapses rare variants across various MAF categories and performs a joint analysis of rare variants in common diseases [43]. The VT method accommodates both trait-increasing and -decreasing variants based on permutation testing with variable allele-frequency thresholds in missense variants [44]. SKAT, a commonly used non-burden test, aggregates rare variants within a region using a new kernel function and identifies trait associations through the variance component score statistic [45]. Notably, the power of the SKAT method increases when causal variants exhibit opposite effects on the trait. For identifying both trait-increasing and -decreasing effects, the SKATO, which is based on the SKAT, was developed [46]. We found that *FRMPD3* (*P* = 4.00 × 10^−6^) was significantly associated with MDD in both functional categories, as shown in Table 2. For the “nonsynonymous” category, *POLA1*, and *FRMPD3* were significantly found in “MAF ≤ 1%” with the following significance thresholds: *P* < 4.60 × 10^−6^ (0.05 / 8782 genes); and *AR*, *FAM47C*, and *ZAN* genes were significantly found in “MAF ≤ 0.1%” with the following significance threshold: *P* < 4.60 × 10^−6^ (0.05 / 10,872 genes) (Supplementary Table S3). For the “damaging” category, *RTL9* and *FRMPD3* genes were significantly found in “MAF ≤ 0.1%” with the following significance threshold: *P* < 1.19 × 10^−5^ (0.05 / 4201 genes) (Supplementary Table S3).

**Table 2:** Results of gene-based rare variants tests.

Gene	Nonsynonymous		Damaging	
	MAF ≤ 0.1%	MAF ≤ 1%	MAF ≤ 0.1%	MAF ≤ 1%
*FRMPD3*	**4.00** × **10**^**−6**^	3.27 × 10^−2^	5.20 × 10^−5^	**4.00** × **10**^**−6**^
Number of genes	8782	10872	3017	4201
*P*-value threshold	5.69 × 10^−6^	4.60 × 10^−6^	1.66 × 10^−5^	1.19 × 10^−5^

After adjusting for the number of genes in each group, significant *P*-values are presented in bold-face.

We used the ACAT method for *P*-values, which first converts the *P*-values from the CMC, SKAT, SKATO, and VT methods to Cauchy variables, and then uses their weighted sum as the test statistic to analyze significance.

The four categories were MAF (≤0.1% and ≤1%) and functional prediction (non-synonymous and damaging variants).

*MAF* minor allele frequency, *ACAT* aggregated Cauchy association test, *CMC* combined multivariate and collapsing, *SKAT* sequence kernel association test, *SKATO* optimized SKAT, *VT* variable threshold, *FRMPD3* FERM and PDZ domains containing 3.

### Neuroimaging-genetic association analysis

DTI parameters of white matter tracts were compared between 234 patients with MDD and 135 HCs, but differences in DTI parameters for white matter tracts were not significant after Benjamini–Hochberg correction (adjusted *P* > 0.05) (Supplementary Table S4).

In the additional analyses, we performed Pearson’s partial correlation analysis between disease burden-related variables (i.e., illness durations and HDRS scores) and DTI parameters, including age, sex, education years, and illness durations/HDRS scores within the MDD group. Illness durations showed significant negative correlations with the FA of both the CCG (left: *r* = −0.260, adjusted *P* = 0.001; right: *r* = −0.223, adjusted *P* = 0.004) and CST (left: *r* = −0.151, adjusted *P* = 0.037; right: *r* = −0.255, adjusted *P* = 0.001), and positive correlations with the RD and MD of several white matter tracts, which were all significant after Benjamini–Hochberg correction (Supplementary Table S5). Additionally, HDRS scores showed significant negative correlations with the AD, MD, and RD of the white matter tracts within the MDD group after Benjamini–Hochberg correction (Supplementary Table S6). Notably, it has been suggested that FA could be a summary measure of microstructural white matter integrity and reflect the number and size of axon fibers [64, 65]. In contrast, MD has been suggested as a marker that is sensitive to cellularity, edema, and necrosis; RD has been suggested to be sensitive to demyelination; and AD may be sensitive to axonal pathologies related to the white matter tissue microstructure and connectivity [64, 65].

We explored the association between neuroimaging markers and SNPs in patients with MDD and HCs. A total of 85 SNPs, to be included in the neuroimaging genetic analysis, were extracted from the following analyses using the present sample: (1) significant variants from the case-control association test in the sample (*P* < 0.001) (30 SNPs); and (2) variants in the most frequently mutated genes (>20% in the patients) in the MDD genome (55 SNPs), which are listed in Supplementary Table S7. The mean and standard deviations of the read depths for the reference and alternative alleles of 85 SNPs are provided in Supplementary Table S7. To correct for multiple testing, we applied the Bonferroni correction with the following significance threshold: *P* < 8.17 × 10^−6^ (0.05 / [4 DTI parameters × 18 white matter tracts × 85 SNPs]) for the white matter tracts.

The SNP rs771995197 in *MUC6* was identified as having a significant effect on DTI parameters following Bonferroni correction (Table 3 and Supplementary Table S8). Specifically, G allele carriers were associated with increased MD and RD in several white matter tracts, including ATR, CCG, CST, FMajor, FMinor, ILF, SLFP, SLFT, and UNC, in combined samples of patients with MDD and HCs. However, we did not detect any significant SNPs with genotype-by-diagnosis interactions.

**Table 3:** Single nucleotide polymorphisms that are significantly associated with white matter tracts in patients with major depressive disorder and healthy controls.

SNP / White matter tracts	Mean	SD	Mean	SD	Genotype		Diagnosis*Genotype	
					*F*	*P*-value	*F*	*P*-value
***MUC6*** rs771995197 (chr11: 1,016,916)*	**AA (*n*** = **288)**		**AG** + **GG (*n*** = **81)**					
MD LH CST	7.30 × 10^−4^	4.51 × 10^−5^	7.71 × 10^−4^	5.92 × 10^−5^	42.52	**2.34** × **10**^**−10**^	0.85	0.358
MD LH SLFP	7.52 × 10^−4^	4.19 × 10^−5^	7.89 × 10^−4^	5.53 × 10^−5^	40.69	**5.41** × **10**^**−10**^	3.19	0.075
RD LH SLFP	5.69 × 10^−4^	4.09 × 10^−5^	6.05 × 10^−4^	5.11 × 10^−5^	40.50	**5.92** × **10**^**−10**^	3.79	0.052
RD LH CST	4.85 × 10^−4^	4.70 × 10^−5^	5.26 × 10^−4^	6.19 × 10^−5^	39.44	**9.63** × **10**^**−10**^	0.86	0.354
MD RH ATR	7.39 × 10^−4^	3.96 × 10^−5^	7.71 × 10^−4^	5.34 × 10^−5^	35.83	**5.15** × **10**^**−9**^	0.90	0.344
MD RH CST	7.10 × 10^−4^	5.22 × 10^−5^	7.52 × 10^−4^	7.05 × 10^−5^	35.44	**6.20** × **10**^**−9**^	0.30	0.584
RD RH CST	4.70 × 10^−4^	5.15 × 10^−5^	5.12 × 10^−4^	6.85 × 10^−5^	34.91	**7.94** × **10**^**−9**^	0.56	0.453
MD RH SLFT	7.31 × 10^−4^	4.32 × 10^−5^	7.67 × 10^−4^	5.72 × 10^−5^	34.89	**8.00** × **10**^**−9**^	0.05	0.819
RD RH SLFP	5.42 × 10^−4^	4.40 × 10^−5^	5.78 × 10^−4^	5.77 × 10^−5^	34.55	**9.41** × **10**^**−9**^	0.34	0.559
RD RH UNC	5.53 × 10^−4^	4.75 × 10^−5^	5.91 × 10^−4^	6.28 × 10^−5^	34.50	**9.64** × **10**^**−9**^	0.45	0.505
MD RH SLFP	7.27 × 10^−4^	4.49 × 10^−5^	7.64 × 10^−4^	6.08 × 10^−5^	33.57	**1.49** × **10**^**−8**^	0.14	0.707
RD RH ATR	5.59 × 10^−4^	3.92 × 10^−5^	5.90 × 10^−4^	5.01 × 10^−5^	33.36	**1.64** × **10**^**−8**^	1.62	0.204
RD RH SLFT	5.35 × 10^−4^	3.87 × 10^−5^	5.66 × 10^−4^	5.21 × 10^−5^	33.35	**1.65** × **10**^**−8**^	0.05	0.822
MD LH ATR	7.44 × 10^−4^	4.20 × 10^−5^	7.76 × 10^−4^	5.13 × 10^−5^	32.80	**2.14** × **10**^**−8**^	0.85	0.358
MD RH CCG	7.26 × 10^−4^	4.41 × 10^−5^	7.60 × 10^−4^	5.84 × 10^−5^	31.68	**3.64** × **10**^**−8**^	0.01	0.937
RD RH CCG	4.67 × 10^−4^	6.03 × 10^−5^	5.14 × 10^−4^	8.54 × 10^−5^	30.54	**6.24** × **10**^**−8**^	0.39	0.533
RD LH ATR	5.63 × 10^−4^	4.23 × 10^−5^	5.92 × 10^−4^	4.80 × 10^−5^	28.75	**1.47** × **10**^**−7**^	1.65	0.200
MD LH CCG	7.34 × 10^−4^	4.69 × 10^−5^	7.67 × 10^−4^	5.94 × 10^−5^	28.08	**2.02** × **10**^**−7**^	0.05	0.820
AD LH SLFP	1.12 × 10^−3^	5.41 × 10^−5^	1.16 × 10^−3^	7.45 × 10^−5^	27.91	**2.19** × **10**^**−7**^	1.55	0.214
AD LH CST	1.22 × 10^−3^	5.74 × 10^−5^	1.26 × 10^−3^	6.70 × 10^−5^	27.89	**2.21** × **10**^−**7**^	0.45	0.501
RD LH SLFT	5.52 × 10^−4^	4.12 × 10^−5^	5.82 × 10^−4^	4.87 × 10^−5^	27.65	**2.48** × **10**^−**7**^	1.72	0.191
AD RH SLFT	1.12 × 10^−3^	6.24 × 10^−5^	1.17 × 10^−3^	7.39 × 10^−5^	27.61	**2.53** × **10**^−**7**^	0.04	0.841
MD RH UNC	7.51 × 10^−4^	5.05 × 10^−5^	7.86 × 10^−4^	6.14 × 10^−5^	26.90	**3.56** × **10**^−**7**^	0.13	0.723
AD RH CST	1.19 × 10^−3^	6.66 × 10^−5^	1.23 × 10^−3^	8.13 × 10^−5^	25.71	**6.33** × **10**^−**7**^	0.02	0.884
RD FMinor	5.48 × 10^−4^	4.23 × 10^−5^	5.77 × 10^−4^	5.26 × 10^−5^	25.29	**7.76** × **10**^−**7**^	0.12	0.726
MD FMinor	7.79 × 10^−4^	4.05 × 10^−5^	8.08 × 10^−4^	5.23 × 10^−5^	25.18	**8.18** × **10**^−**7**^	0.07	0.794
MD LH SLFT	7.56 × 10^−4^	4.26 × 10^−5^	7.85 × 10^−4^	5.09 × 10^−5^	24.96	**9.12** × **10**^−**7**^	1.84	0.176
AD LH ATR	1.11 × 10^−3^	5.44 × 10^−5^	1.14 × 10^−3^	6.89 × 10^−5^	24.61	**1.08** × **10**^−**6**^	0.04	0.847
MD LH UNC	7.90 × 10^−4^	4.06 × 10^−5^	8.17 × 10^−4^	4.92 × 10^−5^	24.15	**1.35** × **10**^−**6**^	0.03	0.855
MD RH ILF	7.87 × 10^−4^	4.73 × 10^−5^	8.18 × 10^−4^	5.83 × 10^−5^	23.88	**1.54** × **10**^−**6**^	0.07	0.794
AD RH SLFP	1.10 × 10^−3^	5.63 × 10^−5^	1.13 × 10^−3^	7.38 × 10^−5^	23.73	**1.66** × **10**^−**6**^	0.00	0.993
AD RH ATR	1.10 × 10^−3^	5.51 × 10^−5^	1.13 × 10^−3^	7.31 × 10^−5^	23.50	**1.85** × **10**^−**6**^	0.08	0.773
MD FMajor	7.78 × 10^−4^	4.27 × 10^−5^	8.17 × 10^−4^	1.10 × 10^−4^	22.77	**2.65** × **10**^−**6**^	8.67	0.003
RD LH UNC	5.93 × 10^−4^	4.34 × 10^−5^	6.19 × 10^−4^	4.88 × 10^−5^	21.29	**5.48** × **10**^−**6**^	0.85	0.356

White matter tracts with significant genotypes are presented (*P* < 0.05).

Bonferroni correction was used as follows: *P* < 0.05 / (4 DTI parameters × 18 white matter tracts × 85 SNPs) = 8.17 × 10^−6^.

Significant genotypes in white matter tracts after Bonferroni correction are shown in bold-face. (*P* < 8.17 × 10^−6^) * UCSC GRCh38/hg38.

*SNP* single nucleotide polymorphisms, *SD* standard deviation, *F* degree of freedom, *MUC6* Mucin 6, *FMajor* forceps major, *FMinor* forceps minor of the corpus callosum, *ATR* the anterior thalamic radiation, *CCG* cingulum cingulate gyrus bundle, *CST* corticospinal tract, *ILF* inferior longitudinal fasciculus, *SLFP* superior longitudinal fasciculus-parietal bundle, *SLFT* superior longitudinal fasciculus-temporal bundle, *UNC* uncinate fasciculus, *FA* fractional anisotropy, *RD* radial diffusivity, *MD* mean diffusivity, *AD* axial diffusivity, *LH* left hemisphere, *RH* right hemisphere.

## Discussion

In the present study, we identified 40 MDD-related genes with rare exonic missense variants in 367 patients with MDD. Among them, *XIRP2*, *MUC5B*, and *FASN* were frequently mutated in >20% of patients with MDD. Notably, we discovered a novel gene, *FRMPD3*, in which the burden of rare variants was concentrated in patients with MDD. Furthermore, rs771995197 in *MUC6* showed a significant correlation with microstructural changes in extensive white matter tracts in the neuroimaging-genetic analysis. Additionally, we observed 17 recurrent CNAs that were annotated to MDD-related genes, such as a gain on 16q22.1 and a loss on 7q11.21 in the MDD genome.

Our first main finding demonstrated that the novel gene, *FRMPD3*, had a significant impact on patients with MDD and was observed to be significant in the “nonsynonymous” and “damaging” categories. *FRMPD3*, an unexplored homolog of *FRMPD4*, acts as a scaffolding molecule involved in the regulation of dendritic spine morphogenesis by associating with post-synaptic density protein (PSD)-95 [66]. The overexpression of PSD-95 in hippocampal neuronal cells enhances the maturation of glutamatergic synapses; therefore, PSD-95 plays a pivotal role in regulating synaptic maturation, indicating its involvement in stabilizing and modulating synaptic plasticity [67]. Several studies have shown that the disruption of PSD-95 in depression inhibits the production of nNOS-derived free radicals and reduces excitotoxicity by blocking the signaling of calcium-ion-activated N-methyl-D-aspartate receptors in the amygdala [68, 69]. Furthermore, *FRMPD3* is an NPAS4-regulated inhibitory neuronal gene, suggesting that the activity of *FRMPD3* promotes the development of excitatory synaptic connections in somatostatin neurons [70]. Several studies have suggested that NPAS4 may regulate depression, anxiety, and neurocognitive disorders and play a critical role in the correlation between long-term stress and symptoms of depression [71, 72]. Our analysis suggests that *FRMPD3* is involved in synaptic formation and regulation, potentially influencing MDD development by modulating synaptic plasticity. Therefore, although the present study did not investigate the association between SNPs in *FRMPD3* and DTI parameters, there is a possibility that genetic variants in this gene may affect individuals’ predisposition to MDD by leading to white matter microstructural abnormalities. Further studies are required on this issue.

In the neuroimaging-genetic analysis, we observed a significant genotype effect of mucin 6 (*MUC6*) rs771995197 on several DTI parameters. Notably, no significant effects of genotype-by-diagnosis interaction were observed. In particular, the G allele of *MUC6* rs771995197 was associated with widespread impairment of the white matter tract integrity concerning RD and MD in the ATR, CCG, CST, FMajor, FMinor, ILF, SLFP, SLFT, and UNC. These results suggest that the *MUC6* gene rs771995197 may be responsible for changes in the DTI parameters, mainly an increase in the RD and MD of several white matter tracts, which are intermediate neuroimaging phenotypes of depression [26]. Importantly, this effect appears consistent across both the MDD and HC groups, reflecting an undifferentiated impact regardless of MDD diagnosis.

Despite the lack of a significant genotype-by-diagnosis interaction effect, the above SNP was associated with MDD in an association test and depression-related neuroimaging phenotypes. This is similar to our previous combined WES-neuroimaging study, in which one SNP in *CDH23* had a genotype effect on cortical thickness but no genotype-by-diagnosis interaction effect [32]. Notably, a previous WES study suggested that the variable number tandem repeat (VNTR) region of *MUC6* is associated with late-onset Alzheimer’s disease [73], and a GWAS of Finnish twins found that a missense variant in *MUC6* is associated with nicotine addiction [74]. Additionally, *MUC6* has been reported to be associated with the neurotrophin signaling pathway through NFκB1 [74, 75], and this may be a possible neurobiological pathway between *MUC6* rs771995197 and alterations of the white matter integrity.

Higher RD in the white matter tracts, which is one of the most common DTI findings in MDD [26], can serve as a marker of demyelination [76]. Given the vital role of neurotrophins in demyelinating pathologies [77], *MUC6* may be involved in depression-related changes in the structural connectivity of white matter tracts through its involvement in neurotrophin signaling and demyelination pathways. To the best of our knowledge, no previous study has demonstrated a causal relationship between this novel SNP and microstructural changes in the white matter tracts. However, further studies are required to elucidate this postulated mechanism.

By comparing the results of the present study with those of our previous WES study combined with structural neuroimaging analysis in patients with MDD [32], we discovered several genes that were not identified in previous studies, such as *XIRP2*, *MUC5B*, *FRMPD3*, and *MUC6*. Furthermore, a previous study found a significant association between one SNP (rs11592462) of *CDH23* and thinning of the right anterior cingulate cortex, whereas the present study did not find any association between *CDH23* and DTI parameters. This discrepancy might be due to the different sample sizes of the two studies; the present study had double the sample size of the previous studies with regard to WES (367 MDD and 161 HCs vs. 184 MDD and 82 HCs) and genetic neuroimaging analysis (234 MDD and 135 HCs vs. 91 MDD and 75 HCs). Future studies with larger sample sizes are needed to obtain more robust results.

We have identified 17 regions with recurrently altered copy numbers containing MDD-related genes. The most frequently detected CNA regions, involving MDD-related genes, included a copy number gain on 16q22.1 (19%) and copy number losses on 7q11.21 (28%) and 22q11.23 (23%). A recent study reported that copy number gains on 16q22.1–q22.2 are related to headache and anxiety disorders [78]. Another WES study found that copy number loss on 7q11.21 was associated with schizophrenia [79]. Copy number loss on 22q11.23 was detected in schizophrenia, autism spectrum disorder, intellectual disability, anxiety, and depression [80]. Our discovery of MDD-related CNAs in 16q22.1 and 7q11.21 and their associations with MDD is novel. However, additional studies are required to confirm these findings.

The present study had several limitations. First, our study might have limited statistical power to detect gene-based rare variants owing to the relatively small sample size, particularly when compared to recent WES studies conducted with larger population-based samples using the UK Biobank [81, 82]. However, to our knowledge, this case-control study represents the largest MDD sample size using WES to investigate neuroimaging genetic variants in Asians. Second, our findings regarding the association between *MUC6* rs771995197 and the white matter tracts were not validated in independent samples, which may have introduced some uncertainty into our results. Third, we did not present results from a validation set and a predictive model based on identified markers from genetic and neuroimaging analyses. The validation of these markers necessitates samples of the same ancestry [83–85]. However, we encountered challenges in recruiting a sufficient number of participants with the same ancestry. Further investigations in independent groups with similar ancestries are necessary to validate the identified markers and establish a predictive model using these markers. Fourth, no significant differences were observed in the DTI parameters between the MDD and HC groups. However, disease burden-related variables, such as illness duration and HDRS scores, demonstrated a significant association with lower FA or higher RD and MD within the MDD group, aligning with the findings from previous DTI studies on MDD [86, 87]. Despite these associations, the absence of diagnostic effects on the DTI parameters may limit our results in the context of neuroimaging-genetic association analysis. Finally, the MDD group had significantly more years of education than the HC group in the present study. Thus, although all neuroimaging-related analyses included years of education as a covariate of no interest, we cannot exclude the possibility that this may affect our results from the neuroimaging analyses.

In summary, we identified 918 rare exonic missense variants associated with MDD, including *XIRP2*, *MUC5B*, *FASN*, *CDH23*, *MYH13*, *TRIO*, *UNC13D*, *CACNA1B*, and *CACNA1C*, using WES. In the joint analyses of WES and neuroimaging data, one significant SNP (rs771995197) in *MUC6* was associated with microstructural changes in the widespread white matter tracts in patients with MDD and HCs. We also investigated rare variants associated with MDD at the gene level and found that *FRMPD3* was significantly associated with MDD. To our knowledge, this is the first study to discover the associations between white matter tracts and SNPs using WES in Korean patients with MDD. Thus, the current study provides a comprehensive understanding of the genetic impact of rare variants, as well as the influence of genetic components on neurostructural alterations in MDD.

MDD is a highly heterogeneous and complex mental disorder. It has been suggested that the integration of knowledge on brain network dysfunction and genomics may uncover intermediate neuroimaging endophenotypes that provide deep insight into the biotyping of heterogeneous patients with MDD [88]. Therefore, the novel approach combining WES and brain structural alterations used in the present study may reveal several intermediate neuroimaging phenotypes with high heritability and related genetic risk variants that may be used in the classification of heterogeneous patients into neuroscience-based depression subtypes. We believe that these efforts may help introduce the concept of precision psychiatry for MDD.

### Supplementary information

Supplementary materials

Supplementary Table S2

## Data Availability

More detailed or raw data from the current study are available from the corresponding author upon reasonable request.
