# Supplementary material for: Integration of whole-exome sequencing and structural neuroimaging analysis in major depressive disorder: a joint study
Source: Transl Psychiatry. 2024 Mar 9;14:141. doi: 10.1038/s41398-024-02849-4 (PMC10924915; doi:10.1038/s41398-024-02849-4)
Supplement: Supplementary file 1 — Supplementary materials [file 41398_2024_2849_MOESM1_ESM.docx]

**Supplementary Materials**

***MRI data acquisition***

Diffusion tensor images were obtained using an echo-planar imaging sequence with the following parameters: repetition time, 6300 ms; echo time, 84 ms; field of view, 230 mm; 128 × 128 matrix; 3 mm slice thickness with no gap; transversal orientation; voxel size, 1.8 mm × 1.8 mm × 3.0 mm; diffusion directions, 20; number of B0 images, 1; number of slices, 50; b-values, 0 and 600 s/mm^2^; acceleration factor (iPAT- GRAPPA), 2 with 38 reference lines for phase encoding direction and a 6/8-phase partial Fourier. Artifacts in the images derived from the MRI system or head motion were visually checked after MRI scanning. If any artifacts were observed, the participant’s was rescanned with MRI.

**Supplementary Tables**

| **Table S1. List of 44 MDD-related genes selected in the current study.** | | |
| --- | --- | --- |
| Num | Gene | Reference |
| 1 | *COQ8A* | Kato, T., 2015. Whole genome/exome sequencing in mood and psychotic disorders. Psychiatry Clin. Neurosci. 69, 65-76. |
| 2 | *ANO8* | Wong, M.L., Arcos-Burgos, M., Liu, S., Velez, J.I., Yu, C., Baune, B.T., Jawahar, M.C., Arolt, V., Dannlowski, U., Chuah, A., Huttley, G.A., Fogarty, R., Lewis, M.D., Bornstein, S.R., Licinio, J., 2017. The PHF21B gene is associated with major depression and modulates the stress response. Mol. Psychiatr. 22, 1015-1025. |
| 3 | *SLC25A4** | Kato, T., 2015. Whole genome/exome sequencing in mood and psychotic disorders. Psychiatry Clin. Neurosci. 69, 65-76. |
| 4 | *ARHGAP8** | Wong, M.L., Arcos-Burgos, M., Liu, S., Velez, J.I., Yu, C., Baune, B.T., Jawahar, M.C., Arolt, V., Dannlowski, U., Chuah, A., Huttley, G.A., Fogarty, R., Lewis, M.D., Bornstein, S.R., Licinio, J., 2017. The PHF21B gene is associated with major depression and modulates the stress response. Mol. Psychiatr. 22, 1015-1025. |
| 5 | *BCAR3* | Wong, M.L., Arcos-Burgos, M., Liu, S., Velez, J.I., Yu, C., Baune, B.T., Jawahar, M.C., Arolt, V., Dannlowski, U., Chuah, A., Huttley, G.A., Fogarty, R., Lewis, M.D., Bornstein, S.R., Licinio, J., 2017. The PHF21B gene is associated with major depression and modulates the stress response. Mol. Psychiatr. 22, 1015-1025. |
| 6 | *BMP5* | Kato, T., 2015. Whole genome/exome sequencing in mood and psychotic disorders. Psychiatry Clin. Neurosci. 69, 65-76. |
| 7 | *BDNF* | Kishi, T., Yoshimura, R., Ikuta, T., Iwata, N., 2017. Brain-derived neurotrophic factor and major depressive disorder: Evidence from meta-analyses. Front. Psychiatry. 8, 308. |
| 8 | *MAB21L4* | Wong, M.L., Arcos-Burgos, M., Liu, S., Velez, J.I., Yu, C., Baune, B.T., Jawahar, M.C., Arolt, V., Dannlowski, U., Chuah, A., Huttley, G.A., Fogarty, R., Lewis, M.D., Bornstein, S.R., Licinio, J., 2017. The PHF21B gene is associated with major depression and modulates the stress response. Mol. Psychiatr. 22, 1015-1025. |
| 9 | *CNTNAP1* | Wong, M.L., Arcos-Burgos, M., Liu, S., Velez, J.I., Yu, C., Baune, B.T., Jawahar, M.C., Arolt, V., Dannlowski, U., Chuah, A., Huttley, G.A., Fogarty, R., Lewis, M.D., Bornstein, S.R., Licinio, J., 2017. The PHF21B gene is associated with major depression and modulates the stress response. Mol. Psychiatr. 22, 1015-1025. |
| 10 | *ADGRE2* | Wong, M.L., Arcos-Burgos, M., Liu, S., Velez, J.I., Yu, C., Baune, B.T., Jawahar, M.C., Arolt, V., Dannlowski, U., Chuah, A., Huttley, G.A., Fogarty, R., Lewis, M.D., Bornstein, S.R., Licinio, J., 2017. The PHF21B gene is associated with major depression and modulates the stress response. Mol. Psychiatr. 22, 1015-1025. |
| 11 | *TAFA5* | Kim, S., Webster, M.J., 2011. Integrative genome-wide association analysis of cytoarchitectural abnormalities in the prefrontal cortex of psychiatric disorders. Mol. Psychiatry. 16, 452-461. |
| 12 | *FASN* | Wong, M.L., Arcos-Burgos, M., Liu, S., Velez, J.I., Yu, C., Baune, B.T., Jawahar, M.C., Arolt, V., Dannlowski, U., Chuah, A., Huttley, G.A., Fogarty, R., Lewis, M.D., Bornstein, S.R., Licinio, J., 2017. The PHF21B gene is associated with major depression and modulates the stress response. Mol. Psychiatr. 22, 1015-1025. |
| 13 | *HOMER3* | Wong, M.L., Arcos-Burgos, M., Liu, S., Velez, J.I., Yu, C., Baune, B.T., Jawahar, M.C., Arolt, V., Dannlowski, U., Chuah, A., Huttley, G.A., Fogarty, R., Lewis, M.D., Bornstein, S.R., Licinio, J., 2017. The PHF21B gene is associated with major depression and modulates the stress response. Mol. Psychiatr. 22, 1015-1025. |
| 14 | *KRBA1* | Wong, M.L., Arcos-Burgos, M., Liu, S., Velez, J.I., Yu, C., Baune, B.T., Jawahar, M.C., Arolt, V., Dannlowski, U., Chuah, A., Huttley, G.A., Fogarty, R., Lewis, M.D., Bornstein, S.R., Licinio, J., 2017. The PHF21B gene is associated with major depression and modulates the stress response. Mol. Psychiatr. 22, 1015-1025. |
| 15 | *LHPP* | CONVERGE consortium, 2015. Sparse whole-genome sequencing identifies two loci for major depressive disorder. Nature. 523, 588-591. |
| 16 | *LILRA1* | Wong, M.L., Arcos-Burgos, M., Liu, S., Velez, J.I., Yu, C., Baune, B.T., Jawahar, M.C., Arolt, V., Dannlowski, U., Chuah, A., Huttley, G.A., Fogarty, R., Lewis, M.D., Bornstein, S.R., Licinio, J., 2017. The PHF21B gene is associated with major depression and modulates the stress response. Mol. Psychiatr. 22, 1015-1025. |
| 17 | *MUC5B* | Wong, M.L., Arcos-Burgos, M., Liu, S., Velez, J.I., Yu, C., Baune, B.T., Jawahar, M.C., Arolt, V., Dannlowski, U., Chuah, A., Huttley, G.A., Fogarty, R., Lewis, M.D., Bornstein, S.R., Licinio, J., 2017. The PHF21B gene is associated with major depression and modulates the stress response. Mol. Psychiatr. 22, 1015-1025. |
| 18 | *MYH13* | Wong, M.L., Arcos-Burgos, M., Liu, S., Velez, J.I., Yu, C., Baune, B.T., Jawahar, M.C., Arolt, V., Dannlowski, U., Chuah, A., Huttley, G.A., Fogarty, R., Lewis, M.D., Bornstein, S.R., Licinio, J., 2017. The PHF21B gene is associated with major depression and modulates the stress response. Mol. Psychiatr. 22, 1015-1025. |
| 19 | *NKPD1* | Amin, N., Belonogova, N.M., Jovanova, O., Brouwer, R.W., Van Rooij, J.G., Van den Hout, M.C., Svishcheva, G.R., Kraaij, R., Zorkoltseva, I.V., Kirichenko, A.V., Hofman, A., Uitterlinden, A.G., Van, I.W.F., Tiemeier, H., Axenovich, T.I., Van Duijn, C.M., 2017a. Nonsynonymous variation in NKPD1 increases depressive symptoms in European populations. Biol. Psychiatry. 81, 702-707. |
| 20 | *OR2T12** | Wong, M.L., Arcos-Burgos, M., Liu, S., Velez, J.I., Yu, C., Baune, B.T., Jawahar, M.C., Arolt, V., Dannlowski, U., Chuah, A., Huttley, G.A., Fogarty, R., Lewis, M.D., Bornstein, S.R., Licinio, J., 2017. The PHF21B gene is associated with major depression and modulates the stress response. Mol. Psychiatr. 22, 1015-1025. |
| 21 | *ORAI1* | Wong, M.L., Arcos-Burgos, M., Liu, S., Velez, J.I., Yu, C., Baune, B.T., Jawahar, M.C., Arolt, V., Dannlowski, U., Chuah, A., Huttley, G.A., Fogarty, R., Lewis, M.D., Bornstein, S.R., Licinio, J., 2017. The PHF21B gene is associated with major depression and modulates the stress response. Mol. Psychiatr. 22, 1015-1025. |
| 22 | *POLG* | Kato, T., 2015. Whole genome/exome sequencing in mood and psychotic disorders. Psychiatry Clin. Neurosci. 69, 65-76. |
| 23 | *PRR5* | Wong, M.L., Arcos-Burgos, M., Liu, S., Velez, J.I., Yu, C., Baune, B.T., Jawahar, M.C., Arolt, V., Dannlowski, U., Chuah, A., Huttley, G.A., Fogarty, R., Lewis, M.D., Bornstein, S.R., Licinio, J., 2017. The PHF21B gene is associated with major depression and modulates the stress response. Mol. Psychiatr. 22, 1015-1025. |
| 24 | *RRM2B* | Kato, T., 2015. Whole genome/exome sequencing in mood and psychotic disorders. Psychiatry Clin. Neurosci. 69, 65-76. |
| 25 | *SIRT1* | CONVERGE consortium, 2015. Sparse whole-genome sequencing identifies two loci for major depressive disorder. Nature. 523, 588-591. |
| 26 | *SLC2A8* | Wong, M.L., Arcos-Burgos, M., Liu, S., Velez, J.I., Yu, C., Baune, B.T., Jawahar, M.C., Arolt, V., Dannlowski, U., Chuah, A., Huttley, G.A., Fogarty, R., Lewis, M.D., Bornstein, S.R., Licinio, J., 2017. The PHF21B gene is associated with major depression and modulates the stress response. Mol. Psychiatr. 22, 1015-1025. |
| 27 | *TMEM150B* | Wong, M.L., Arcos-Burgos, M., Liu, S., Velez, J.I., Yu, C., Baune, B.T., Jawahar, M.C., Arolt, V., Dannlowski, U., Chuah, A., Huttley, G.A., Fogarty, R., Lewis, M.D., Bornstein, S.R., Licinio, J., 2017. The PHF21B gene is associated with major depression and modulates the stress response. Mol. Psychiatr. 22, 1015-1025. |
| 28 | *TRIO* | Wong, M.L., Arcos-Burgos, M., Liu, S., Velez, J.I., Yu, C., Baune, B.T., Jawahar, M.C., Arolt, V., Dannlowski, U., Chuah, A., Huttley, G.A., Fogarty, R., Lewis, M.D., Bornstein, S.R., Licinio, J., 2017. The PHF21B gene is associated with major depression and modulates the stress response. Mol. Psychiatr. 22, 1015-1025. |
| 29 | *TWNK* | Kato, T., 2015. Whole genome/exome sequencing in mood and psychotic disorders. Psychiatry Clin. Neurosci. 69, 65-76. |
| 30 | *UNC13D* | Wong, M.L., Arcos-Burgos, M., Liu, S., Velez, J.I., Yu, C., Baune, B.T., Jawahar, M.C., Arolt, V., Dannlowski, U., Chuah, A., Huttley, G.A., Fogarty, R., Lewis, M.D., Bornstein, S.R., Licinio, J., 2017. The PHF21B gene is associated with major depression and modulates the stress response. Mol. Psychiatr. 22, 1015-1025. |
| 31 | *RCL1* | Amin, N., De Vrij, F.M.S., Baghdadi, M., Brouwer, R.W.W., Van Rooij, J.G.J., Jovanova, O., Uitterlinden, A.G., Hofman, A., Janssen, H.L.A., Darwish Murad, S., Kraaij, R., Stedehouder, J., Van den Hout, M., Kros, J.M., Van, I.W.F.J., Tiemeier, H., Kushner, S.A., Van Duijn, C.M., 2018. A rare missense variant in RCL1 segregates with depression in extended families. Mol. Psychiatr. 23, 1120-1126. |
| 32 | *LIPG* | Amin, N., Jovanova, O., Adams, H.H., Dehghan, A., Kavousi, M., Vernooij, M.W., Peeters, R.P., De Vrij, F.M., Van der Lee, S.J., Van Rooij, J.G., Van Leeuwen, E.M., Chaker, L., Demirkan, A., Hofman, A., Brouwer, R.W., Kraaij, R., Willems van Dijk, K., Hankemeier, T., Van Ijcken, W.F., Uitterlinden, A.G., Niessen, W.J., Franco, O.H., Kushner, S.A., Ikram, M.A., Tiemeier, H., Van Duijn, C.M., 2017b. Exome-sequencing in a large population-based study reveals a rare Asn396Ser variant in the LIPG gene associated with depressive symptoms. Mol. Psychiatr. 22, 537-543. |
| 33 | *CACNA1B* | Tombacz, D., Maroti, Z., Kalmar, T., Csabai, Z., Balazs, Z., Takahashi, S., Palkovits, M., Snyder, M., Boldogkoi, Z., 2017. High-coverage whole-exome sequencing identifies candidate genes for suicide in victims with major depressive disorder. Sci. Rep. 7, 7106. |
| 34 | *CACNA1C* | Tombacz, D., Maroti, Z., Kalmar, T., Csabai, Z., Balazs, Z., Takahashi, S., Palkovits, M., Snyder, M., Boldogkoi, Z., 2017. High-coverage whole-exome sequencing identifies candidate genes for suicide in victims with major depressive disorder. Sci. Rep. 7, 7106. |
| 35 | *CACNA2D4* | Tombacz, D., Maroti, Z., Kalmar, T., Csabai, Z., Balazs, Z., Takahashi, S., Palkovits, M., Snyder, M., Boldogkoi, Z., 2017. High-coverage whole-exome sequencing identifies candidate genes for suicide in victims with major depressive disorder. Sci. Rep. 7, 7106. |
| 36 | *CDH23* | Han, K. M., Han, M. R., Kim, A., Kang, W., Kang, Y., Kang, J., Tae, W. S., Cho, Y., & Ham, B. J., 2020. A study combining whole-exome sequencing and structural neuroimaging analysis for major depressive disorder. Journal of affective disorders, 262, 31–39. |
| 37 | *SYT8* | Zhu, S., He, M., Liu, Z., Qin, Z., Wang, Z., & Duan, L.,2020. Shared genetic susceptibilities for irritable bowel syndrome and depressive disorder in Chinese patients uncovered by pooled whole-exome sequencing. Journal of advanced research, 23, 113–121. |
| 38 | *SSPO** | Zhu, S., He, M., Liu, Z., Qin, Z., Wang, Z., & Duan, L.,2020. Shared genetic susceptibilities for irritable bowel syndrome and depressive disorder in Chinese patients uncovered by pooled whole-exome sequencing. Journal of advanced research, 23, 113–121. |
| 39 | *KRTAP1-1* | Park, J. H., Lim, S. W., Myung, W., Park, I., Jang, H. J., Kim, S., Lee, M. S., Chang, H. S., Yum, D., Suh, Y. L., Kim, J. W., & Kim, D. K., 2021. Whole-genome sequencing reveals KRTAP1-1 as a novel genetic variant associated with antidepressant treatment outcomes. Scientific reports, 11(1), 4552. |
| 40 | *XIRP2* | Liu, Y., Qu, H. Q., Chang, X., Qu, J., Mentch, F. D., Nguyen, K., Tian, L., Glessner, J., Sleiman, P., & Hakonarson, H., 2022. Mutation Burden Analysis of Six Common Mental Disorders in African Americans by Whole Genome Sequencing. Human molecular genetics, ddac129. Advance online publication. |
| 41 | *NPPC* | Liu, Y., Qu, H. Q., Chang, X., Qu, J., Mentch, F. D., Nguyen, K., Tian, L., Glessner, J., Sleiman, P., & Hakonarson, H., 2022. Mutation Burden Analysis of Six Common Mental Disorders in African Americans by Whole Genome Sequencing. Human molecular genetics, ddac129. Advance online publication. |
| 42 | *STK33* | Liu, Y., Qu, H. Q., Chang, X., Qu, J., Mentch, F. D., Nguyen, K., Tian, L., Glessner, J., Sleiman, P., & Hakonarson, H., 2022. Mutation Burden Analysis of Six Common Mental Disorders in African Americans by Whole Genome Sequencing. Human molecular genetics, ddac129. Advance online publication. |
| 43 | *PANX1* | Liu, Y., Qu, H. Q., Chang, X., Qu, J., Mentch, F. D., Nguyen, K., Tian, L., Glessner, J., Sleiman, P., & Hakonarson, H., 2022. Mutation Burden Analysis of Six Common Mental Disorders in African Americans by Whole Genome Sequencing. Human molecular genetics, ddac129. Advance online publication. |
| 44 | *NTS* | Liu, Y., Qu, H. Q., Chang, X., Qu, J., Mentch, F. D., Nguyen, K., Tian, L., Glessner, J., Sleiman, P., & Hakonarson, H., 2022. Mutation Burden Analysis of Six Common Mental Disorders in African Americans by Whole Genome Sequencing. Human molecular genetics, ddac129. Advance online publication. |
| * Signifies a gene mutation that was NOT identified in the current study. | | |

**Table S3. Gene-based rare variants test results (MDD versus HCs)**

| **Nonsynonymous** |  |  |
| --- | --- | --- |
| **Gene** | **MAF ≤ 1%** | **MAF ≤ 0.1%** |
| **AR** | 6.95E-02 | **4.00E-06** |
| NKTR | 1.53E-01 | 1.52E-01 |
| THEG | 1.11E-01 | 7.60E-05 |
| MED12 | 6.81E-02 | 1.01E-01 |
| OR2W3 | 4.30E-01 | 1.08E-01 |
| UGT1A4 | 1.95E-01 | 3.20E-04 |
| ALMS1 | 2.19E-03 | 9.60E-03 |
| **FAM47C** | 1.24E-01 | **4.00E-06** |
| PRRG3 | 5.96E-02 | 5.72E-02 |
| UGT1A8 | 1.97E-01 | 1.10E-01 |
| UGT1A10 | 4.43E-02 | 4.52E-04 |
| IRS4 | 1.67E-01 | 1.92E-04 |
| BCAM | 6.78E-02 | 7.25E-02 |
| **POLA1** | **4.00E-06** | 2.15E-01 |
| RTL9 | 1.07E-01 | 8.41E-02 |
| **FRMPD3** | **4.00E-06** | 3.27E-02 |
| **ZAN** | 3.40E-02 | **4.00E-06** |
| Number of genes | 8782 | 10872 |
| P-value threshold | 5.69E-06 | 4.60E-06 |
| **Damaging** |  |  |
| **Gene** | **MAF ≤ 1%** | **MAF ≤ 0.1%** |
| FAAH2 | 5.55E-02 | 4.15E-02 |
| ABCA2 | 4.75E-03 | 9.47E-03 |
| MAGEB4 | 1.91E-01 | 5.49E-03 |
| OR2T27 | 7.87E-03 | 1.08E-02 |
| **RTL9** | 1.21E-01 | **6.00E-06** |
| **FRMPD3** | 5.20E-05 | **4.00E-06** |
| Number of genes | 3017 | 4201 |
| P-value threshold | 1.66E-05 | 1.19E-05 |

After adjusting for the number of genes in each group, P-values in bold were considered significant. We use the ACAT method for P-values that first converts P-values (CMC, SKAT, SKATO, and VT) to Cauchy variables, and then uses their weighted sum as the test statistic to analyze significance. Four categories resulting from filtering of the minor allele frequency (MAF) and functional prediction were included.

MAF, minor allele frequency; ACAT, aggregated Cauchy association test; MDD, major depressive disorder; HC, healthy controls.

**Table S4. Comparisons of integrity of white matter tracts between patients with MDD and HCs**

| **White matter tracts** | **MDD (n = 234)** | | **HC (n = 135)** | | **F(1, 363)** | **P-value** | **Adjusted P-value** |
| --- | --- | --- | --- | --- | --- | --- | --- |
|  | **Mean** | **SD** | **Mean** | **SD** |  |  |  |
|  |  |  |  |  |  |  |  |
| AD LH CCG | 1.25E-03 | 7.23E-05 | 1.27E-03 | 9.43E-05 | 7.939 | 0.005 | 0.105 |
| AD LH CST | 1.22E-03 | 5.91E-05 | 1.24E-03 | 6.56E-05 | 7.684 | 0.006 | 0.105 |
| MD LH CST | 7.36E-04 | 5.09E-05 | 7.44E-04 | 5.17E-05 | 7.691 | 0.006 | 0.105 |
| MD RH SLFT | 7.36E-04 | 4.72E-05 | 7.45E-04 | 5.14E-05 | 7.809 | 0.005 | 0.105 |
| AD RH SLFT | 1.13E-03 | 6.36E-05 | 1.14E-03 | 7.34E-05 | 6.770 | 0.010 | 0.116 |
| RD RH SLFT | 5.40E-04 | 4.42E-05 | 5.46E-04 | 4.33E-05 | 7.067 | 0.008 | 0.116 |
| AD RH CCG | 1.24E-03 | 6.64E-05 | 1.26E-03 | 5.54E-05 | 5.383 | 0.021 | 0.120 |
| AD RH CST | 1.20E-03 | 6.77E-05 | 1.21E-03 | 7.97E-05 | 5.072 | 0.025 | 0.120 |
| AD RH SLFP | 1.10E-03 | 5.69E-05 | 1.11E-03 | 7.08E-05 | 6.068 | 0.014 | 0.120 |
| RD LH CST | 4.92E-04 | 5.45E-05 | 4.98E-04 | 5.09E-05 | 5.691 | 0.018 | 0.120 |
| RD LH SLFP | 5.76E-04 | 4.91E-05 | 5.80E-04 | 3.92E-05 | 5.394 | 0.021 | 0.120 |
| RD RH CST | 4.77E-04 | 5.75E-05 | 4.83E-04 | 5.94E-05 | 5.122 | 0.024 | 0.120 |
| RD RH SLFP | 5.48E-04 | 4.94E-05 | 5.53E-04 | 4.97E-05 | 5.221 | 0.023 | 0.120 |
| MD RH CST | 7.16E-04 | 5.65E-05 | 7.24E-04 | 6.39E-05 | 5.757 | 0.017 | 0.120 |
| MD RH SLFP | 7.33E-04 | 4.87E-05 | 7.40E-04 | 5.48E-05 | 6.163 | 0.013 | 0.120 |
| MD LH SLFP | 7.59E-04 | 4.90E-05 | 7.62E-04 | 4.54E-05 | 4.547 | 0.034 | 0.151 |
| FA RH CAB | 4.01E-01 | 5.47E-02 | 3.94E-01 | 4.73E-02 | 3.696 | 0.055 | 0.210 |
| RD LH SLFT | 5.58E-04 | 4.77E-05 | 5.61E-04 | 3.85E-05 | 3.869 | 0.050 | 0.210 |
| MD LH CCG | 7.40E-04 | 5.11E-05 | 7.43E-04 | 5.30E-05 | 3.761 | 0.053 | 0.210 |
| FA LH UNC | 4.28E-01 | 3.30E-02 | 4.26E-01 | 3.15E-02 | 3.277 | 0.071 | 0.256 |
| AD RH ILF | 1.26E-03 | 6.96E-05 | 1.26E-03 | 7.77E-05 | 3.136 | 0.077 | 0.265 |
| MD LH SLFT | 7.62E-04 | 4.81E-05 | 7.64E-04 | 4.25E-05 | 2.808 | 0.095 | 0.310 |
| MD RH ILF | 7.92E-04 | 5.28E-05 | 7.96E-04 | 4.95E-05 | 2.603 | 0.108 | 0.337 |
| MD RH CCG | 7.32E-04 | 4.88E-05 | 7.36E-04 | 5.11E-05 | 2.501 | 0.115 | 0.344 |
| AD LH SLFP | 1.12E-03 | 5.94E-05 | 1.13E-03 | 6.49E-05 | 2.289 | 0.131 | 0.378 |
| FA LH ILF | 4.77E-01 | 3.15E-02 | 4.85E-01 | 2.69E-02 | 2.181 | 0.141 | 0.389 |
| FA RH UNC | 4.47E-01 | 3.40E-02 | 4.46E-01 | 3.26E-02 | 2.114 | 0.147 | 0.392 |
| FA LH SLFT | 4.63E-01 | 2.70E-02 | 4.63E-01 | 2.24E-02 | 1.799 | 0.181 | 0.434 |
| AD LH ILF | 1.26E-03 | 6.72E-05 | 1.27E-03 | 6.86E-05 | 1.859 | 0.174 | 0.434 |
| RD RH UNC | 5.61E-04 | 5.24E-05 | 5.62E-04 | 5.57E-05 | 1.830 | 0.177 | 0.434 |
| AD RH ATR | 1.10E-03 | 5.71E-05 | 1.11E-03 | 6.84E-05 | 1.633 | 0.202 | 0.469 |
| RD FMinor | 5.55E-04 | 4.64E-05 | 5.54E-04 | 4.63E-05 | 1.461 | 0.228 | 0.497 |
| RD RH ILF | 5.61E-04 | 5.35E-05 | 5.62E-04 | 4.16E-05 | 1.477 | 0.225 | 0.497 |
| FA LH SLFP | 4.32E-01 | 2.87E-02 | 4.31E-01 | 2.10E-02 | 1.310 | 0.253 | 0.525 |
| FA RH CST | 5.45E-01 | 3.57E-02 | 5.45E-01 | 3.03E-02 | 1.300 | 0.255 | 0.525 |
| FA FMajor | 5.84E-01 | 4.70E-02 | 5.92E-01 | 2.53E-02 | 0.930 | 0.336 | 0.554 |
| FA FMinor | 4.82E-01 | 3.79E-02 | 4.83E-01 | 3.76E-02 | 0.937 | 0.334 | 0.554 |
| FA LH CST | 5.39E-01 | 3.52E-02 | 5.40E-01 | 2.98E-02 | 0.923 | 0.337 | 0.554 |
| AD LH SLFT | 1.17E-03 | 5.88E-05 | 1.17E-03 | 5.97E-05 | 0.918 | 0.339 | 0.554 |
| AD LH UNC | 1.19E-03 | 5.17E-05 | 1.18E-03 | 6.58E-05 | 1.097 | 0.296 | 0.554 |
| RD LH ATR | 5.71E-04 | 4.77E-05 | 5.67E-04 | 4.07E-05 | 0.953 | 0.330 | 0.554 |
| RD LH UNC | 5.99E-04 | 4.84E-05 | 5.98E-04 | 4.14E-05 | 1.076 | 0.300 | 0.554 |
| MD LH ATR | 7.52E-04 | 4.59E-05 | 7.50E-04 | 4.65E-05 | 1.033 | 0.310 | 0.554 |
| MD RH ATR | 7.47E-04 | 4.45E-05 | 7.44E-04 | 4.61E-05 | 0.922 | 0.338 | 0.554 |
| FA RH ATR | 4.19E-01 | 3.22E-02 | 4.25E-01 | 2.42E-02 | 0.771 | 0.380 | 0.595 |
| AD FMinor | 1.25E-03 | 6.84E-05 | 1.24E-03 | 7.57E-05 | 0.790 | 0.375 | 0.595 |
| AD LH ATR | 1.12E-03 | 5.56E-05 | 1.11E-03 | 6.68E-05 | 0.741 | 0.390 | 0.597 |
| AD FMajor | 1.40E-03 | 8.76E-05 | 1.40E-03 | 6.66E-05 | 0.604 | 0.438 | 0.657 |
| MD RH UNC | 7.59E-04 | 5.29E-05 | 7.58E-04 | 5.87E-05 | 0.575 | 0.449 | 0.659 |
| FA LH CCG | 5.53E-01 | 5.29E-02 | 5.62E-01 | 3.36E-02 | 0.326 | 0.568 | 0.760 |
| RD LH CCG | 4.85E-04 | 6.23E-05 | 4.79E-04 | 4.38E-05 | 0.359 | 0.549 | 0.760 |
| RD RH ATR | 5.68E-04 | 4.57E-05 | 5.63E-04 | 3.98E-05 | 0.327 | 0.568 | 0.760 |
| RD RH CAB | 5.96E-04 | 1.41E-04 | 5.95E-04 | 8.42E-05 | 0.323 | 0.570 | 0.760 |
| RD RH CCG | 4.78E-04 | 7.14E-05 | 4.76E-04 | 6.58E-05 | 0.399 | 0.528 | 0.760 |
| FA LH CAB | 3.76E-01 | 4.54E-02 | 3.78E-01 | 5.16E-02 | 0.213 | 0.645 | 0.844 |
| MD LH ILF | 8.04E-04 | 5.12E-05 | 8.02E-04 | 4.39E-05 | 0.199 | 0.656 | 0.844 |
| FA RH CCG | 5.50E-01 | 6.44E-02 | 5.58E-01 | 5.23E-02 | 0.103 | 0.749 | 0.896 |
| AD LH CAB | 1.15E-03 | 1.32E-04 | 1.14E-03 | 1.12E-04 | 0.051 | 0.822 | 0.896 |
| AD RH CAB | 1.11E-03 | 1.57E-04 | 1.10E-03 | 1.33E-04 | 0.126 | 0.723 | 0.896 |
| AD RH UNC | 1.16E-03 | 6.91E-05 | 1.15E-03 | 7.70E-05 | 0.063 | 0.802 | 0.896 |
| RD FMajor | 4.85E-04 | 8.42E-05 | 4.74E-04 | 4.01E-05 | 0.056 | 0.813 | 0.896 |
| RD LH CAB | 6.38E-04 | 1.09E-04 | 6.34E-04 | 8.85E-05 | 0.056 | 0.813 | 0.896 |
| RD LH ILF | 5.76E-04 | 5.07E-05 | 5.70E-04 | 3.92E-05 | 0.091 | 0.763 | 0.896 |
| MD FMinor | 7.87E-04 | 4.39E-05 | 7.83E-04 | 4.64E-05 | 0.112 | 0.739 | 0.896 |
| MD LH UNC | 7.97E-04 | 4.43E-05 | 7.93E-04 | 4.35E-05 | 0.061 | 0.805 | 0.896 |
| MD RH CAB | 7.67E-04 | 1.42E-04 | 7.62E-04 | 9.51E-05 | 0.053 | 0.817 | 0.896 |
| FA LH ATR | 4.19E-01 | 3.15E-02 | 4.23E-01 | 2.33E-02 | 0.020 | 0.887 | 0.927 |
| FA RH SLFT | 4.58E-01 | 2.64E-02 | 4.61E-01 | 2.00E-02 | 0.020 | 0.888 | 0.927 |
| MD FMajor | 7.89E-04 | 7.58E-05 | 7.82E-04 | 4.33E-05 | 0.022 | 0.881 | 0.927 |
| FA RH SLFP | 4.45E-01 | 2.94E-02 | 4.48E-01 | 2.48E-02 | 0.005 | 0.942 | 0.963 |
| MD LH CAB | 8.08E-04 | 1.12E-04 | 8.02E-04 | 8.94E-05 | 0.004 | 0.950 | 0.963 |
| FA RH ILF | 4.90E-01 | 3.54E-02 | 4.92E-01 | 2.55E-02 | 0.001 | 0.979 | 0.979 |

The mean and standard deviation (SD) of the DTI parameters of white matter tracts are shown.

BH correction was performed for the white matter tracts.

The analysis of covariance comprised the covariates of age, sex, and years of education.

MDD, major depressive disorder; HC, healthy controls; F, degree of freedom; SD, standard deviation; FMajor, forceps major; FMinor, forceps minor of the corpus callosum; ATR, the anterior thalamic radiation; CAB, cingulum-angular bundle; CCG, cingulum cingulate gyrus bundle; CST, corticospinal tract; ILF, inferior longitudinal fasciculus; SLFP, superior longitudinal fasciculus-parietal bundle; SLFT, superior longitudinal fasciculus-temporal bundle; UNC, uncinate fasciculus; FA, fractional anisotropy; RD, radial diffusivity; MD, mean diffusivity; AD, axial diffusivity; LH, left hemisphere; RH, right hemisphere.

**Table S5. Correlation analysis between illness duration and integrity of the white matter tracts in patients with MDD**

| **WMT** | **r** | **P-value** | **Adjusted P-value** |
| --- | --- | --- | --- |
| FA RH CST | -.255 | 3.55 × 10^-5^ | 0.001 |
| MD RH ILF | .254 | 3.69 × 10^-5^ | 0.001 |
| RD LH CCG | .253 | 4.83 × 10^-5^ | 0.001 |
| FA LH CCG | -.260 | 5.19 × 10^-5^ | 0.001 |
| RD RH CST | .238 | 5.45 × 10^-5^ | 0.001 |
| RD RH ILF | .241 | 9.92 × 10^-5^ | 0.001 |
| RD RH CCG | .235 | 1.31 × 10^-4^ | 0.001 |
| FA RH CCG | -.223 | 3.97 × 10^-4^ | 0.004 |
| MD RH CST | .203 | 0.001 | 0.004 |
| AD RH ILF | .203 | 0.001 | 0.008 |
| AD FMinor | .196 | 0.001 | 0.008 |
| RD RH SLFP | .178 | 0.003 | 0.016 |
| MD RH UNC | .182 | 0.003 | 0.016 |
| RD LH CST | .170 | 0.003 | 0.016 |
| MD RH CCG | .179 | 0.003 | 0.016 |
| MD LH CCG | .171 | 0.004 | 0.019 |
| RD RH UNC | .171 | 0.005 | 0.023 |
| MD RH SLFP | .156 | 0.007 | 0.029 |
| MD LH CST | .149 | 0.008 | 0.031 |
| AD RH SLFT | .148 | 0.009 | 0.031 |
| MD RH SLFT | .144 | 0.011 | 0.037 |
| AD RH UNC | .157 | 0.011 | 0.037 |
| FA LH CST | -.151 | 0.012 | 0.037 |
| MD LH CAB | .153 | 0.013 | 0.039 |
| RD LH CAB | .148 | 0.016 | 0.047 |
| AD LH CAB | .147 | 0.018 | 0.049 |
| MD RH ATR | .137 | 0.020 | 0.054 |
| MD LH UNC | .145 | 0.021 | 0.055 |
| RD LH SLFP | .136 | 0.023 | 0.058 |
| MD FMajor | .135 | 0.025 | 0.060 |
| FA RH SLFP | -.137 | 0.032 | 0.071 |
| AD RH CAB | .133 | 0.033 | 0.071 |
| MD LH ATR | .127 | 0.034 | 0.071 |
| RD RH SLFT | .123 | 0.035 | 0.071 |
| FA FMinor | .130 | 0.037 | 0.071 |
| RD LH UNC | .131 | 0.037 | 0.071 |
| RD RH ATR | .126 | 0.037 | 0.071 |
| MD RH CAB | .128 | 0.038 | 0.071 |
| AD LH ATR | .120 | 0.039 | 0.071 |
| AD FMajor | .122 | 0.039 | 0.071 |
| RD LH ILF | .129 | 0.042 | 0.074 |
| AD RH ATR | .116 | 0.045 | 0.077 |
| FA LH SLFT | -.127 | 0.052 | 0.084 |
| RD RH CAB | .118 | 0.053 | 0.084 |
| RD FMajor | .119 | 0.054 | 0.084 |
| AD LH UNC | .123 | 0.054 | 0.084 |
| RD LH SLFT | .111 | 0.055 | 0.085 |
| MD LH SLFP | .107 | 0.058 | 0.087 |
| MD LH ILF | .118 | 0.059 | 0.087 |
| FA LH SLFP | -.126 | 0.062 | 0.090 |
| AD RH CST | .104 | 0.069 | 0.097 |
| RD LH ATR | .111 | 0.072 | 0.100 |
| MD FMinor | .104 | 0.085 | 0.115 |
| AD RH SLFP | .093 | 0.096 | 0.129 |
| FA RH ILF | -.107 | 0.099 | 0.130 |
| AD RH CCG | -.104 | 0.121 | 0.156 |
| FA RH CAB | -.089 | 0.142 | 0.179 |
| MD LH SLFT | .080 | 0.149 | 0.185 |
| FA LH ILF | -.092 | 0.167 | 0.203 |
| FA FMajor | -.085 | 0.179 | 0.215 |
| AD LH CST | .071 | 0.190 | 0.224 |
| AD LH ILF | .075 | 0.226 | 0.262 |
| FA LH CAB | -.072 | 0.259 | 0.296 |
| FA RH UNC | -.071 | 0.266 | 0.299 |
| AD LH CCG | -.071 | 0.352 | 0.389 |
| AD LH SLFP | .039 | 0.381 | 0.416 |
| FA RH ATR | -.052 | 0.445 | 0.478 |
| FA LH UNC | -.048 | 0.465 | 0.492 |
| AD LH SLFT | .014 | 0.685 | 0.714 |
| FA RH SLFT | -.017 | 0.826 | 0.849 |
| FA LH ATR | -.015 | 0.865 | 0.877 |
| RD FMinor | -.001 | 0.975 | 0.975 |

Pearson’s correlation analysis included age, sex, years of education, and HDRS scores as covariates.

The adjusted P-values were derived using the Benjamini–Hochberg correction.

FMajor, forceps major; FMinor, forceps minor of the corpus callosum; ATR, anterior thalamic radiation; CAB, cingulum-angular bundle; CCG, cingulum cingulate gyrus bundle; CST, corticospinal tract; ILF, inferior longitudinal fasciculus; SLFP, superior longitudinal fasciculus-parietal bundle; SLFT, superior longitudinal fasciculus-temporal bundle; UNC, uncinate fasciculus; FA, fractional anisotropy; RD, radial diffusivity; MD, mean diffusivity; AD, axial diffusivity; LH, left hemisphere; RH, right hemisphere.

**Table S6. Correlation analysis between HDRS scores and integrity of the white matter tracts in patients with MDD**

| **WMT** | **r** | **P-value** | **Adjusted P-value** |
| --- | --- | --- | --- |
| AD LH SLFP | -.247 | 1.58 × 10^-4^ | 0.009 |
| MD LH UNC | -.240 | 2.40 × 10^-4^ | 0.009 |
| MD LH SLFP | -.218 | 8.56 × 10^-4^ | 0.015 |
| AD LH UNC | -.216 | 9.85 × 10^-4^ | 0.015 |
| AD LH SLFT | -.213 | 0.001 | 0.015 |
| RD LH UNC | -.211 | 0.001 | 0.015 |
| AD LH ILF | -.199 | 0.002 | 0.026 |
| MD LH SLFT | -.190 | 0.004 | 0.031 |
| AD RH SLFP | -.190 | 0.004 | 0.031 |
| MD LH ILF | -.185 | 0.005 | 0.034 |
| MD RH UNC | -.184 | 0.005 | 0.034 |
| MD RH SLFP | -.173 | 0.009 | 0.046 |
| AD FMajor | -.172 | 0.009 | 0.046 |
| RD LH SLFP | -.172 | 0.009 | 0.046 |
| RD RH UNC | -.169 | 0.010 | 0.049 |
| AD FMinor | -.167 | 0.011 | 0.049 |
| AD LH CST | -.165 | 0.012 | 0.049 |
| AD RH UNC | -.165 | 0.012 | 0.049 |
| RD LH SLFT | -.153 | 0.020 | 0.077 |
| RD LH CAB | -.150 | 0.023 | 0.082 |
| MD RH ILF | -.149 | 0.024 | 0.082 |
| RD LH ILF | -.146 | 0.026 | 0.084 |
| AD RH SLFT | -.145 | 0.028 | 0.084 |
| MD LH CST | -.144 | 0.029 | 0.084 |
| RD RH SLFP | -.144 | 0.029 | 0.084 |
| MD LH ATR | -.140 | 0.034 | 0.093 |
| MD RH ATR | -.137 | 0.037 | 0.093 |
| MD LH CAB | -.137 | 0.037 | 0.093 |
| RD LH ATR | -.137 | 0.037 | 0.093 |
| AD RH ILF | -.136 | 0.039 | 0.093 |
| MD RH SLFT | -.132 | 0.045 | 0.105 |
| RD RH ILF | -.128 | 0.052 | 0.117 |
| AD RH CST | -.126 | 0.057 | 0.125 |
| FA LH CAB | .123 | 0.063 | 0.130 |
| AD RH ATR | -.123 | 0.063 | 0.130 |
| RD RH ATR | -.122 | 0.065 | 0.131 |
| MD FMinor | -.118 | 0.073 | 0.143 |
| RD LH CST | -.110 | 0.096 | 0.183 |
| AD LH ATR | -.107 | 0.104 | 0.193 |
| RD RH SLFT | -.105 | 0.112 | 0.200 |
| AD LH CAB | -.104 | 0.115 | 0.200 |
| FA LH ATR | .103 | 0.118 | 0.200 |
| AD LH CCG | -.103 | 0.120 | 0.200 |
| MD RH CST | -.101 | 0.126 | 0.207 |
| RD RH CCG | -.100 | 0.130 | 0.209 |
| MD RH CCG | -.098 | 0.137 | 0.215 |
| FA LH UNC | .094 | 0.155 | 0.237 |
| MD FMajor | -.091 | 0.167 | 0.251 |
| MD LH CCG | -.087 | 0.189 | 0.277 |
| FA RH CCG | .076 | 0.252 | 0.359 |
| FA FMajor | -.075 | 0.254 | 0.359 |
| FA RH UNC | .074 | 0.262 | 0.362 |
| RD RH CST | -.073 | 0.270 | 0.366 |
| FA FMinor | -.069 | 0.299 | 0.399 |
| FA RH ILF | .063 | 0.342 | 0.447 |
| FA RH SLFP | .058 | 0.383 | 0.492 |
| FA LH CST | .049 | 0.458 | 0.579 |
| FA RH ATR | .047 | 0.483 | 0.599 |
| RD LH CCG | -.045 | 0.502 | 0.612 |
| RD FMinor | -.043 | 0.517 | 0.621 |
| FA LH ILF | .040 | 0.543 | 0.641 |
| FA RH CAB | .038 | 0.567 | 0.659 |
| RD FMajor | -.032 | 0.633 | 0.723 |
| FA RH SLFT | .028 | 0.672 | 0.756 |
| FA LH SLFT | .019 | 0.770 | 0.853 |
| FA RH CST | .015 | 0.824 | 0.876 |
| FA LH SLFP | .015 | 0.825 | 0.876 |
| FA LH CCG | -.014 | 0.827 | 0.876 |
| AD RH CAB | -.009 | 0.889 | 0.928 |
| AD RH CCG | -.005 | 0.937 | 0.964 |
| MD RH CAB | -.002 | 0.975 | 0.976 |
| RD RH CAB | .002 | 0.976 | 0.976 |

Pearson’s correlation analysis included age, sex, years of education, and HDRS scores as covariates.

The adjusted P-values were derived using the Benjamini–Hochberg correction.

FMajor, forceps major; FMinor, forceps minor of the corpus callosum; ATR, anterior thalamic radiation; CAB, cingulum-angular bundle; CCG, cingulum cingulate gyrus bundle; CST, corticospinal tract; ILF, inferior longitudinal fasciculus; SLFP, superior longitudinal fasciculus-parietal bundle; SLFT, superior longitudinal fasciculus-temporal bundle; UNC, uncinate fasciculus; FA, fractional anisotropy; RD, radial diffusivity; MD, mean diffusivity; AD, axial diffusivity; LH, left hemisphere; RH, right hemisphere.

| **Table S7. List of candidate single nucleotide polymorphism (SNPs) for neuroimaging-genetic association analysis** | | | | | | | | |
| --- | --- | --- | --- | --- | --- | --- | --- | --- |
| Num | | Gene | SNP | Genomic position (GRCh38) | Ref | Alt | Read depth of ref (Mean ± SD) | Read depth of alt (Mean ± SD) |
| 1 | *SPATA21* | | rs41269193 | Chr1:16403813 | G | T | 48.72±14.32 | 11.06±27.36 |
| 2 | *TMCO4* | | rs4515815 | Chr1:19694501 | C | T | 52.23±22.14 | 25.82±32.33 |
| 3 | *MAP3K21* | | rs189326455 | Chr1:233328190 | G | C | 9.56±7.21 | 0.48±2.03 |
| 4 | *SLC39A12* | | rs2478568 | Chr10:17978060 | G | A | 23.66±26.56 | 68.43±37.13 |
| 5 | *ZFAND4* | | rs80042909 | Chr10:45626603 | C | T | 39.11±10.88 | 5.31±18.06 |
| 6 | *CDH23* | | rs1227049 | Chr10:71675131 | G | C | 29.47±12.11 | 15.19±15.77 |
| 7 | *CDH23* | | rs10999947 | Chr10:71675149 | G | A | 29.35±11.29 | 13.75±15.94 |
| 8 | *CDH23* | | rs1227065 | Chr10:71732322 | A | G | 28.83±45.34 | 139.36±54 |
| 9 | *CDH23* | | rs56181447 | Chr10:71738598 | G | A | 41.41±15.7 | 8.62±15.78 |
| 10 | *CDH23* | | rs1227051 | Chr10:71741799 | G | A | 28.41±43.15 | 147.55±57.7 |
| 11 | *CDH23* | | rs17712523 | Chr10:71777857 | G | A | 90.33±33.99 | 18.88±33.8 |
| 12 | *CDH23* | | rs3802711 | Chr10:71784329 | G | A | 39.23±13.23 | 18.89±26.61 |
| 13 | *CDH23* | | rs11592462 | Chr10:71790360 | C | G | 67.87±22.79 | 18.41±32.7 |
| 14 | *CDH23* | | rs10466026 | Chr10:71791212 | G | A | 32.96±23.21 | 44.34±35.18 |
| 15 | *CDH23* | | rs41281334 | Chr10:71798371 | G | A | 43.87±8.82 | 2.3±9.92 |
| 16 | *CDH23* | | rs4747194 | Chr10:71799129 | G | A | 27.62±18.51 | 33.81±25.33 |
| 17 | *CDH23* | | rs4747195 | Chr10:71799195 | C | T | 50.22±34.01 | 63.38±46.4 |
| 18 | *CDH23* | | rs45583140 | Chr10:71812008 | T | C | 56.97±27.18 | 35.06±46.87 |
| 19 | *UTF1* | | rs11599284 | Chr10:133230505 | G | A | 11.7±5.6 | 1±2.81 |
| 20 | *MUC6* | | rs771995197 | Chr11:1016916 | A | G | 337.56±94.66 | 8.62±16.95 |
| 21 | *MUC5B* | | rs2672785 | Chr11:1225711 | A | G | 28.44±16.59 | 26.43±25.06 |
| 22 | *MUC5B* | | rs2075853 | Chr11:1226228 | C | T | 41.3±18.64 | 30.9±36.12 |
| 23 | *MUC5B* | | rs10835639 | Chr11:1241082 | G | A | 42.5±21.19 | 21.35±23.56 |
| 24 | *MUC5B* | | rs1541314 | Chr11:1242293 | G | A | 76.76±24.63 | 14.85±30.59 |
| 25 | *MUC5B* | | rs2943510 | Chr11:1242546 | C | T | 73.7±26.46 | 11.78±24.74 |
| 26 | *MUC5B* | | rs55813014 | Chr11:1246095 | T | C | 43.16±43.61 | 76.95±54.13 |
| 27 | *MUC5B* | | rs58125533 | Chr11:1246245 | T | C | 72.22±40.01 | 1.68±4.3 |
| 28 | *MUC5B* | | rs117757264 | Chr11:1246440 | G | A | 29.3±13.15 | 1.39±5.1 |
| 29 | *MUC5B* | | rs2943512 | Chr11:1250996 | A | C | 23.06±25.36 | 45.96±35.36 |
| 30 | *MUC5B* | | rs2943511 | Chr11:1251015 | C | T | 64.25±35.38 | 8.02±18.34 |
| 31 | *MUC5B* | | rs3021155 | Chr11:1251479 | G | A | 50.4±24.05 | 5.61±12.58 |
| 32 | *MUC5B* | | rs3021156 | Chr11:1251524 | A | G | 52.26±22.77 | 6.32±13.9 |
| 33 | *MUC5B* | | rs3829224 | Chr11:1255097 | G | A | 35.23±14.23 | 18.3±24.07 |
| 34 | *RESF1* | | rs3759296 | Chr12:31984967 | A | G | 42.64±8.55 | 9.47±19.34 |
| 35 | *PRIM1* | | rs2277339 | Chr12:56752285 | T | G | 34.57±11.52 | 13.77±18.95 |
| 36 | *PABPC3* | | rs115121649 | Chr13:25097621 | C | T | 172.28±47.15 | 3.13±7.59 |
| 37 | *PPCDC* | | rs2304899 | Chr15:75044388 | A | G | 17.16±29.09 | 100.13±39.89 |
| 38 | *SEMA4B* | | rs3751655 | Chr15:90228518 | T | G | 12.1±16.22 | 41.99±19.03 |
| 39 | *TPSB2* | | rs62012862 | Chr16:1228744 | C | T | 5.27±7.27 | 20.09±10.64 |
| 40 | *SMTNL2* | | rs12449695 | Chr17:4592445 | G | A | 10.95±10.43 | 18.31±11.95 |
| 41 | *MYH13* | | rs3744550 | Chr17:10303278 | T | C | 22.92±11.38 | 9.71±10.9 |
| 42 | *MYH13* | | rs17690195 | Chr17:10315796 | C | T | 37±10.56 | 15.65±22.62 |
| 43 | *MYH13* | | rs2074876 | Chr17:10320380 | G | T | 33.21±16.72 | 23.34±22.44 |
| 44 | *MYH13* | | rs2074877 | Chr17:10320397 | T | C | 29.08±18.09 | 30.93±26.5 |
| 45 | *KRTAP1-3* | | rs62622847 | Chr17:41034506 | T | C | 19.25±9.16 | 5.14±7.71 |
| 46 | *ABCA8* | | rs35403175 | Chr17:68875696 | C | T | 40.77±4.96 | 2.43±9.25 |
| 47 | *FASN* | | rs17848945 | Chr17:82082637 | C | T | 27.64±8.16 | 1.36±4.5 |
| 48 | *FASN* | | rs45557233 | Chr17:82084074 | G | A | 42.34±10.14 | 9.1±20.12 |
| 49 | *ZNF610* | | rs2241586 | Chr19:52365769 | G | T | 15.8±12.87 | 20.45±13.84 |
| 50 | *ZNF610* | | rs321937 | Chr19:52366025 | G | C | 18.82±14.47 | 23.65±16.42 |
| 51 | *LILRA1* | | rs28524012 | Chr19:54594257 | G | C | 36.37±15.07 | 10.7±12.25 |
| 52 | *LILRA1* | | rs1974982 | Chr19:54594278 | A | G | 35.67±15.08 | 10.04±11.57 |
| 53 | *LILRA1* | | rs75416770 | Chr19:54595231 | C | T | 92.15±22.17 | 5.21±19.47 |
| 54 | *XIRP2* | | rs77546992 | Chr2:167184577 | C | T | 47.1±8.27 | 3.84±11.65 |
| 55 | *XIRP2* | | rs77278822 | Chr2:167218196 | C | T | 40.35±8.28 | 6.37±15.96 |
| 56 | *XIRP2* | | rs16853305 | Chr2:167243265 | C | G | 66.52±38.48 | 39.4±50.28 |
| 57 | *XIRP2* | | rs16853306 | Chr2:167243286 | T | C | 62.88±33.45 | 47.95±55.41 |
| 58 | *XIRP2* | | rs75802875 | Chr2:167245060 | T | C | 37.62±9.96 | 2.6±7.94 |
| 59 | *XIRP2* | | rs16853309 | Chr2:167246794 | G | A | 63.2±25.39 | 27.44±46.47 |
| 60 | *XIRP2* | | rs77219745 | Chr2:167246908 | G | A | 56.47±15.03 | 8.86±26.73 |
| 61 | *XIRP2* | | rs7607246 | Chr2:167247415 | A | G | 47.28±13.76 | 10.6±18.31 |
| 62 | *XIRP2* | | rs61750760 | Chr2:167248117 | G | A | 52.32±15.08 | 13.2±22.98 |
| 63 | *XIRP2* | | rs59889092 | Chr2:167248478 | G | T | 54.36±20.48 | 9.09±15.88 |
| 64 | *XIRP2* | | rs16853328 | Chr2:167249575 | G | A | 42.22±10.42 | 12.51±21.64 |
| 65 | *XIRP2* | | rs16853330 | Chr2:167249736 | G | A | 47.3±18.58 | 31.13±35.73 |
| 66 | *XIRP2* | | rs3749002 | Chr2:167250100 | G | A | 41.53±6.27 | 3.6±11.3 |
| 67 | *XIRP2* | | rs143084183 | Chr2:167250525 | A | G | 39.93±7.36 | 2.95±9.24 |
| 68 | *XIRP2* | | rs16853331 | Chr2:167250645 | G | A | 54.78±25.36 | 25.85±44.32 |
| 69 | *XIRP2* | | rs3749003 | Chr2:167250841 | A | G | 56.07±21.52 | 12.63±30.77 |
| 70 | *XIRP2* | | rs3749004 | Chr2:167250981 | A | G | 45.33±14.2 | 11.63±20.5 |
| 71 | *XIRP2* | | rs3749005 | Chr2:167258815 | A | G | 34.82±6.47 | 4.67±10.87 |
| 72 | *XIRP2* | | rs3749006 | Chr2:167259200 | T | G | 51.32±26.29 | 36.17±48.78 |
| 73 | *XIRP2* | | rs16853344 | Chr2:167259259 | G | C | 61.14±33.18 | 26.5±45.62 |
| 74 | *ASIC4* | | rs11689281 | Chr2:219537949 | G | T | 8.74±10.79 | 20.81±11.78 |
| 75 | *ASIC4* | | rs11695248 | Chr2:219537958 | T | C | 9.68±11.82 | 23.12±13.3 |
| 76 | *DEFB127* | | rs12624954 | Chr20:158815 | G | A | 44.17±23.23 | 40.86±35.02 |
| 77 | *DEFB127* | | rs16995685 | Chr20:158935 | C | A | 51.8±28.86 | 53.54±45.63 |
| 78 | *SEL1L2* | | rs2073290 | Chr20:13849491 | A | C | 45.83±9.09 | 7.13±16.29 |
| 79 | *C22orf42* | | rs5998267 | Chr22:32158998 | A | G | 27.82±15.38 | 22.15±19.77 |
| 80 | *DCHS2* | | rs61743677 | Chr4:154235863 | T | C | 76.81±21.19 | 15.33±28.05 |
| 81 | *POC5* | | rs17672542 | Chr5:75705757 | A | G | 14±10.29 | 10.16±9.59 |
| 82 | *SPINK5* | | rs2303070 | Chr5:148120328 | G | T | 69.71±28.86 | 32.27±36.13 |
| 83 | *ADAP1* | | rs79805216 | Chr7:955367 | G | C | 31.4±11.98 | 17.05±18.86 |
| 84 | *HSD17B3* | | rs2066479 | Chr9:96235528 | C | T | 53.97±18.59 | 23.9±31.68 |
| 85 | *HMCN2* | | rs11244308 | Chr9:130430524 | G | A | 43±8.56 | 6.87±20.09 |
| Chr, chromosome; Alt, Alternative allele; Ref, Reference allele; SD, Standard deviation; GRCh38, Genome Reference Consortium Human Build 38 | | | | | | | | |

**Table S8. Single nucleotide polymorphisms that are significantly associated with white matter tract integrity in patients with MDD and HCs**

|  |  | | |  |  | Genotype | |  | Diagnosis*Genotype | |
| --- | --- | --- | --- | --- | --- | --- | --- | --- | --- | --- |
| SNP / White matter tracts | Mean | SD | Mean | SD |  | F | P-value |  | F | P-value |
| ***MUC6* rs771995197 (chr11: 1,016,916)*** | **AA (n = 288)** | | **AG+GG (n = 81)** | |  |  |  |  |  |  |
| MD LH CST | 7.30E-04 | 4.51E-05 | 7.71E-04 | 5.92E-05 |  | 42.52 | **2.34E-10** |  | 0.85 | 0.358 |
| MD LH SLFP | 7.52E-04 | 4.19E-05 | 7.89E-04 | 5.53E-05 |  | 40.69 | **5.41E-10** |  | 3.19 | 0.075 |
| RD LH SLFP | 5.69E-04 | 4.09E-05 | 6.05E-04 | 5.11E-05 |  | 40.50 | **5.92E-10** |  | 3.79 | 0.052 |
| RD LH CST | 4.85E-04 | 4.70E-05 | 5.26E-04 | 6.19E-05 |  | 39.44 | **9.63E-10** |  | 0.86 | 0.354 |
| MD RH ATR | 7.39E-04 | 3.96E-05 | 7.71E-04 | 5.34E-05 |  | 35.83 | **5.15E-09** |  | 0.90 | 0.344 |
| MD RH CST | 7.10E-04 | 5.22E-05 | 7.52E-04 | 7.05E-05 |  | 35.44 | **6.20E-09** |  | 0.30 | 0.584 |
| RD RH CST | 4.70E-04 | 5.15E-05 | 5.12E-04 | 6.85E-05 |  | 34.91 | **7.94E-09** |  | 0.56 | 0.453 |
| MD RH SLFT | 7.31E-04 | 4.32E-05 | 7.67E-04 | 5.72E-05 |  | 34.89 | **8.00E-09** |  | 0.05 | 0.819 |
| RD RH SLFP | 5.42E-04 | 4.40E-05 | 5.78E-04 | 5.77E-05 |  | 34.55 | **9.41E-09** |  | 0.34 | 0.559 |
| RD RH UNC | 5.53E-04 | 4.75E-05 | 5.91E-04 | 6.28E-05 |  | 34.50 | **9.64E-09** |  | 0.45 | 0.505 |
| MD RH SLFP | 7.27E-04 | 4.49E-05 | 7.64E-04 | 6.08E-05 |  | 33.57 | **1.49E-08** |  | 0.14 | 0.707 |
| RD RH ATR | 5.59E-04 | 3.92E-05 | 5.90E-04 | 5.01E-05 |  | 33.36 | **1.64E-08** |  | 1.62 | 0.204 |
| RD RH SLFT | 5.35E-04 | 3.87E-05 | 5.66E-04 | 5.21E-05 |  | 33.35 | **1.65E-08** |  | 0.05 | 0.822 |
| MD LH ATR | 7.44E-04 | 4.20E-05 | 7.76E-04 | 5.13E-05 |  | 32.80 | **2.14E-08** |  | 0.85 | 0.358 |
| MD RH CCG | 7.26E-04 | 4.41E-05 | 7.60E-04 | 5.84E-05 |  | 31.68 | **3.64E-08** |  | 0.01 | 0.937 |
| RD RH CCG | 4.67E-04 | 6.03E-05 | 5.14E-04 | 8.54E-05 |  | 30.54 | **6.24E-08** |  | 0.39 | 0.533 |
| RD LH ATR | 5.63E-04 | 4.23E-05 | 5.92E-04 | 4.80E-05 |  | 28.75 | **1.47E-07** |  | 1.65 | 0.200 |
| MD LH CCG | 7.34E-04 | 4.69E-05 | 7.67E-04 | 5.94E-05 |  | 28.08 | **2.02E-07** |  | 0.05 | 0.820 |
| AD LH SLFP | 1.12E-03 | 5.41E-05 | 1.16E-03 | 7.45E-05 |  | 27.91 | **2.19E-07** |  | 1.55 | 0.214 |
| AD LH CST | 1.22E-03 | 5.74E-05 | 1.26E-03 | 6.70E-05 |  | 27.89 | **2.21E-07** |  | 0.45 | 0.501 |
| RD LH SLFT | 5.52E-04 | 4.12E-05 | 5.82E-04 | 4.87E-05 |  | 27.65 | **2.48E-07** |  | 1.72 | 0.191 |
| AD RH SLFT | 1.12E-03 | 6.24E-05 | 1.17E-03 | 7.39E-05 |  | 27.61 | **2.53E-07** |  | 0.04 | 0.841 |
| MD RH UNC | 7.51E-04 | 5.05E-05 | 7.86E-04 | 6.14E-05 |  | 26.90 | **3.56E-07** |  | 0.13 | 0.723 |
| AD RH CST | 1.19E-03 | 6.66E-05 | 1.23E-03 | 8.13E-05 |  | 25.71 | **6.33E-07** |  | 0.02 | 0.884 |
| RD FMinor | 5.48E-04 | 4.23E-05 | 5.77E-04 | 5.26E-05 |  | 25.29 | **7.76E-07** |  | 0.12 | 0.726 |
| MD FMinor | 7.79E-04 | 4.05E-05 | 8.08E-04 | 5.23E-05 |  | 25.18 | **8.18E-07** |  | 0.07 | 0.794 |
| MD LH SLFT | 7.56E-04 | 4.26E-05 | 7.85E-04 | 5.09E-05 |  | 24.96 | **9.12E-07** |  | 1.84 | 0.176 |
| AD LH ATR | 1.11E-03 | 5.44E-05 | 1.14E-03 | 6.89E-05 |  | 24.61 | **1.08E-06** |  | 0.04 | 0.847 |
| MD LH UNC | 7.90E-04 | 4.06E-05 | 8.17E-04 | 4.92E-05 |  | 24.15 | **1.35E-06** |  | 0.03 | 0.855 |
| MD RH ILF | 7.87E-04 | 4.73E-05 | 8.18E-04 | 5.83E-05 |  | 23.88 | **1.54E-06** |  | 0.07 | 0.794 |
| AD RH SLFP | 1.10E-03 | 5.63E-05 | 1.13E-03 | 7.38E-05 |  | 23.73 | **1.66E-06** |  | 0.00 | 0.993 |
| AD RH ATR | 1.10E-03 | 5.51E-05 | 1.13E-03 | 7.31E-05 |  | 23.50 | **1.85E-06** |  | 0.08 | 0.773 |
| MD FMajor | 7.78E-04 | 4.27E-05 | 8.17E-04 | 1.10E-04 |  | 22.77 | **2.65E-06** |  | 8.67 | 0.003 |
| RD LH UNC | 5.93E-04 | 4.34E-05 | 6.19E-04 | 4.88E-05 |  | 21.29 | **5.48E-06** |  | 0.85 | 0.356 |
| FA RH UNC | 4.51E-01 | 3.07E-02 | 4.32E-01 | 3.85E-02 |  | 20.36 | 8.67E-06 |  | 0.37 | 0.541 |
| RD RH ILF | 5.55E-04 | 4.65E-05 | 5.83E-04 | 5.32E-05 |  | 19.84 | 1.12E-05 |  | 0.78 | 0.378 |
| RD RH CAB | 5.80E-04 | 9.41E-05 | 6.48E-04 | 1.86E-04 |  | 19.13 | 1.60E-05 |  | 3.33 | 0.069 |
| FA RH CCG | 5.60E-01 | 5.45E-02 | 5.27E-01 | 7.23E-02 |  | 18.73 | 1.95E-05 |  | 0.80 | 0.373 |
| FA RH CST | 5.49E-01 | 3.23E-02 | 5.31E-01 | 3.50E-02 |  | 18.63 | 2.05E-05 |  | 0.38 | 0.538 |
| RD LH CCG | 4.76E-04 | 5.20E-05 | 5.06E-04 | 6.45E-05 |  | 18.30 | 2.42E-05 |  | 1.23 | 0.268 |
| AD RH ILF | 1.25E-03 | 6.72E-05 | 1.29E-03 | 8.32E-05 |  | 18.29 | 2.42E-05 |  | 0.40 | 0.530 |
| RD FMajor | 4.72E-04 | 4.60E-05 | 5.11E-04 | 1.21E-04 |  | 18.20 | 2.54E-05 |  | 7.58 | 0.006 |
| MD LH ILF | 7.98E-04 | 4.54E-05 | 8.24E-04 | 5.41E-05 |  | 17.79 | 3.12E-05 |  | 0.85 | 0.356 |
| MD RH CAB | 7.51E-04 | 9.80E-05 | 8.17E-04 | 1.88E-04 |  | 17.56 | 3.51E-05 |  | 2.59 | 0.109 |
| FA LH CST | 5.43E-01 | 3.11E-02 | 5.26E-01 | 3.73E-02 |  | 17.20 | 4.20E-05 |  | 0.31 | 0.580 |
| AD LH ILF | 1.25E-03 | 6.38E-05 | 1.29E-03 | 7.41E-05 |  | 17.04 | 4.55E-05 |  | 0.06 | 0.809 |
| AD FMajor | 1.39E-03 | 6.93E-05 | 1.43E-03 | 1.06E-04 |  | 16.36 | 6.41E-05 |  | 5.16 | 0.024 |
| AD LH CCG | 1.25E-03 | 7.55E-05 | 1.29E-03 | 9.38E-05 |  | 16.16 | 7.07E-05 |  | 1.12 | 0.290 |
| AD LH UNC | 1.18E-03 | 5.27E-05 | 1.21E-03 | 6.78E-05 |  | 14.59 | 1.57E-04 |  | 1.03 | 0.311 |
| AD LH SLFT | 1.16E-03 | 5.50E-05 | 1.19E-03 | 6.74E-05 |  | 13.81 | 2.34E-04 |  | 1.39 | 0.240 |
| FA RH SLFP | 4.49E-01 | 2.73E-02 | 4.36E-01 | 2.76E-02 |  | 13.24 | 3.14E-04 |  | 0.02 | 0.878 |
| RD LH ILF | 5.69E-04 | 4.40E-05 | 5.91E-04 | 5.28E-05 |  | 12.55 | 4.49E-04 |  | 1.59 | 0.208 |
| AD RH CAB | 1.09E-03 | 1.26E-04 | 1.16E-03 | 2.02E-04 |  | 11.72 | 6.87E-04 |  | 1.16 | 0.282 |
| MD LH CAB | 7.96E-04 | 1.03E-04 | 8.40E-04 | 1.04E-04 |  | 11.25 | 8.79E-04 |  | 0.06 | 0.806 |
| RD LH CAB | 6.27E-04 | 1.00E-04 | 6.70E-04 | 1.00E-04 |  | 10.97 | 1.02E-03 |  | 0.01 | 0.938 |
| AD RH UNC | 1.15E-03 | 7.04E-05 | 1.18E-03 | 7.35E-05 |  | 10.05 | 1.65E-03 |  | 0.03 | 0.875 |
| FA LH SLFT | 4.65E-01 | 2.42E-02 | 4.55E-01 | 2.78E-02 |  | 9.90 | 1.79E-03 |  | 0.09 | 0.764 |
| FA RH CAB | 4.03E-01 | 5.13E-02 | 3.83E-01 | 5.23E-02 |  | 9.46 | 2.26E-03 |  | 1.30 | 0.256 |
| AD LH CAB | 1.13E-03 | 1.22E-04 | 1.18E-03 | 1.28E-04 |  | 9.10 | 2.74E-03 |  | 0.24 | 0.627 |
| AD FMinor | 1.24E-03 | 6.91E-05 | 1.27E-03 | 7.50E-05 |  | 8.51 | 3.76E-03 |  | 0.81 | 0.367 |
| FA LH SLFP | 4.34E-01 | 2.55E-02 | 4.24E-01 | 2.70E-02 |  | 8.23 | 4.36E-03 |  | 0.71 | 0.399 |
| FA FMajor | 5.90E-01 | 3.65E-02 | 5.76E-01 | 5.12E-02 |  | 6.47 | 1.14E-02 |  | 2.21 | 0.138 |
| FA RH ATR | 4.23E-01 | 2.92E-02 | 4.14E-01 | 3.00E-02 |  | 6.02 | 1.46E-02 |  | 0.43 | 0.513 |
| FA FMinor | 4.85E-01 | 3.73E-02 | 4.73E-01 | 3.80E-02 |  | 5.38 | 2.10E-02 |  | 1.12 | 0.290 |
| FA LH ATR | 4.22E-01 | 2.90E-02 | 4.14E-01 | 2.72E-02 |  | 4.39 | 3.68E-02 |  | 0.85 | 0.359 |
| FA LH UNC | 4.29E-01 | 3.18E-02 | 4.20E-01 | 3.42E-02 |  | 4.33 | 3.81E-02 |  | 2.15 | 0.144 |
| FA RH SLFT | 4.61E-01 | 2.42E-02 | 4.54E-01 | 2.39E-02 |  | 4.23 | 4.03E-02 |  | 0.05 | 0.827 |

White matter tracts with significant genotypes are shown (P < 0.05).

Bonferroni correction was used as follows: P < 0.05 / (4 DTI parameters × 18 white matter tracts × 85 SNPs) = 8.17E-06

Significant genotypes in white matter tracts after Bonferroni correction are shown in bold-face. (P < 8.17E-06)

* UCSC GRCh38/hg38

SNP, single nucleotide polymorphisms; SD, standard deviation; MUC6, Mucin 6; FMajor, forceps major; FMinor, forceps minor of the corpus callosum; ATR, the anterior thalamic radiation; CAB, cingulum-angular bundle; CCG, cingulum cingulate gyrus bundle; CST, corticospinal tract; ILF, inferior longitudinal fasciculus; SLFP, superior longitudinal fasciculus-parietal bundle; SLFT, superior longitudinal fasciculus-temporal bundle; UNC, uncinate fasciculus; FA, fractional anisotropy; RD, radial diffusivity; MD, mean diffusivity; AD, axial diffusivity; LH, left hemisphere; RH, right hemisphere.
